# Shared Determinants of Poor Sleep, Obesity and Adiposity in Adolescents Aged 8–18‐Years: A Systematic Review

**DOI:** 10.1111/jsr.70029

**Published:** 2025-03-10

**Authors:** Emma Louise Gale, Joanne Elizabeth Cecil, Andrew James Williams

**Affiliations:** ^1^ School of Medicine University of St Andrews Scotland UK; ^2^ School of Health in Social Science University of Edinburgh Scotland UK

**Keywords:** behaviours, BMI, chronotype, sleep; obesity; shared determinants, teenagers, wellbeing

## Abstract

Relationships between multiple sleep outcomes, obesity and adiposity across childhood and adolescence have been previously reported. Health‐promoting interventions to improve sleep and reduce adolescent obesity could target shared determinants of sleep and obesity. The aim of this systematic review was to systematically identify and examine research that investigated the shared determinants of poor sleep and increased adiposity or obesity in adolescents. A systematic literature search covering publications up to April 2024 was conducted across 10 bibliographic databases. Search terms included objective and subjective sleep/circadian rhythm measurements, objective adiposity measurements and adolescents aged 8–18 years. Eighty studies were included in the final review. Determinants were categorised into three different domains: socioenvironmental determinants, behavioural determinants and health determinants. Shared determinants of poor sleep and increased adiposity or obesity in adolescents aged 8–18 years included: socioenvironmental determinants (gender, ethnicity, pubertal status, academic attainment), behavioural determinants (timing of moderate‐to‐vigorous physical activity (MVPA), unhealthy diet choices and timing of consumption and screen time and videogaming quantity and timing) and health determinants (wellbeing). These findings suggest that sleep hygiene and the modifiable shared behavioural determinants should be targeted in health‐promoting interventions, and statistical analyses should be adjusted for socioenvironmental determinants and wellbeing.

## 1 Introduction

The interplay between poor sleep and obesity in adolescents underscores a critical public health challenge (Williams et al. 2015; Lim et al. 2023). Adequate sleep plays a pivotal role in the physical and mental wellbeing of adolescents (Tarokh et al. 2016; Liu et al. 2013; Gariépy et al. 2019), and its significance in combating obesity cannot be overemphasised (Miller et al. 2021). According to the WHO, in 2024, 43% of the world's adult population was classified as overweight (body mass index (BMI) ≥ 25), and 16% were classified as living with obesity (BMI ≥ 30), double that reported in 1990 (World Health Organisation 2024). Recent research on the global economic impacts of overweight and obesity has estimated that a reduction of 5% in the projected prevalence of obesity between 2020 and 2060 could reduce annual spending of US$ 429 billion (~£337.5 billion) worldwide; if there were to be a halt in the projected prevalence of overweight and obesity between 2020 and 2060, there would be an annual saving of US$2.20 trillion (~£1.7 trillion) globally (Okunogbe et al. 2022). Globally, in 2024, 20% of children and adolescents were overweight (BMI percentile (BMIp) ≥ 85th), and 8% were living with obesity (BMIp ≥ 95th), compared with 8% overweight and 2% living with obesity in 1990 (World Health Organisation 2024).

Research from previous systematic reviews in adolescents has highlighted relationships between pre‐sleep outcomes (including but not limited to sleep hygiene, sleep habits and insomnia symptoms), during‐sleep outcomes (including sleep quality and efficiency) and sleep timings (later and irregular) with adiposity or obesity in adolescents (Miller et al. 2021; Fatima et al. 2015; Gale et al. 2024; Miller et al. 2018; Morrissey et al. 2020). Adiposity, unlike obesity, is a measure of body fat rather than a derivative measure of height and weight combined (Kim 2016). Additional systematic reviews have reported a bidirectional relationship between sleep duration and obesity in adolescents (Fatima et al. 2015; Morrissey et al. 2020; Fatima et al. 2016). Inadequate sleep disrupts the balance of hormones that regulate appetite and metabolism, increasing the likelihood of weight gain in children and adolescents (Taheri et al. 2004). Poor sleep can contribute to insulin resistance (Johnson et al. 2022; St‐Onge et al. 2023), higher blood sugar levels (Simon et al. 2019; Wang et al. 2023) and increased fat storage (Redinger 2009), which are crucial factors in the development of obesity (Simon et al. 2019; Thota et al. 2017). Moreover, sleep deprivation often results in increased energy intake due to extended waking hours, providing more opportunities to eat, particularly high‐calorie, low‐nutrient foods (He et al. 2015; Jankovic et al. 2023). These combined factors create a significant risk of weight gain and the development of obesity (Vujović et al. 2022). Additionally, insufficient sleep diminishes cognitive function (Lo et al. 2016; De Bruin et al. 2017), exacerbates stress (LaVoy et al. 2020; Hirotsu et al. 2015), impairs decision‐making abilities (Lau et al. 2019; Short and Weber 2018) and increases vulnerability to unhealthy dietary choices and sedentary behaviours (Reilly et al. 2003; Wadden and Stunkard 1985; Banks and Dinges 2007; Medic et al. 2017). The transition to adolescence is a period in which individuals experience a hormonal and growth shift (Sawyer et al. 2018) and the development of a delayed sleep pattern (Tarokh et al. 2016; Crowley et al. 2007). During puberty, adolescents develop new behaviours (e.g., excessive screen time, poorer food choices and less physical activity) due to exploring their new autonomy, some of which can be unhealthy and cause adolescents to become more susceptible to weight gain (Jebeile et al. 2022) and poorer sleep (Owens and Weiss 2017).

As childhood obesity rates continue to rise globally, addressing the pivotal role of sleep in weight management is imperative for safeguarding the long‐term health and wellbeing of children and adolescents (Williams et al. 2015; Sanyaolu et al. 2019). Prioritising strategies to improve sleep among children could be essential in mitigating the obesity epidemic and cultivating healthier futures. One way research could help develop these strategies is to identify specific determinants to be targeted in health‐promoting interventions in adolescents. Previous systematic reviews have examined the individual determinants of sleep and obesity, and adiposity, respectively, in adolescents (Morrissey et al. 2020; Chaput et al. 2016; Li et al. 2017). However, researchers have speculated whether there are shared determinants, given the interconnected mechanisms and relationships between sleep, obesity and adiposity, that could be targeted to enhance the effectiveness of health‐promoting interventions in adolescents (Duraccio et al. 2019; Felső et al. 2017).

The aim of this systematic review was to systematically identify and examine research investigating the determinants of poor sleep and increased adiposity or obesity in adolescents that should be considered when designing a public health intervention for a specific demographic. Specific research objectives included: (i) to identify the shared non‐modifiable (socioenvironmental) determinants of poor sleep and increased obesity in adolescents; (ii) to identify the shared modifiable determinants (behavioural determinants) of poor sleep and increased obesity in adolescents; and (iii) to determine health determinants that co‐exist with poor sleep and increased obesity in adolescents.

## 2 Methods

### 2.1 Literature Search

A search of the following database took place in December 2022, followed by an updated search in April 2024: MEDLINE, EMBASE, Ovid, Web of Science, Scopus, PsychInfo, CINAHL, The Cochrane Library (including Cochrane Database of Systematic Reviews, the Cochrane Central Register of Controlled Trials (CENTRAL) and the Cochrane Methodology Register) and Education Research Information Centre (ERIC). The review followed the Preferred Reporting Items for Systematic Reviews and Meta‐Analyses (PRISMA) 2020 guidelines (Page et al. 2021). There was no restriction on the time of publication or the country where a study was conducted or published. The search terms are shown in Table 1.

**TABLE 1:** List of search terms used in the database search.

Line number	Search term
1	obes* OR overweight OR BMI OR body mass OR weight
2	chronotype OR circadian* OR social jetlag OR sleep* OR (biological adj4 rhythm) OR clock* OR eveningness OR morningness
3	adolescen* OR child* OR young* OR youth* OR teen* OR school* OR student*
4	observational OR prospective OR longitudinal OR cohort OR cross‐sectional OR nested OR intervention OR trial OR clinical study OR RCT OR randomised controlled trial
5	1 AND 2 AND 3 AND 4

### 2.2 Selection of Studies

Studies were filtered for relevance using inclusion and exclusion criteria (Section 2.3). First, study duplicates were removed, and the remaining papers were screened based on title, then abstract and full‐text level according to inclusion/exclusion criteria (Table 2). All database searches and screening of papers at abstract and title level were conducted by one author (EG). Independently, two authors then screened all papers at the full‐text level (EG, AJW), and another author reviewed a sample of potentially relevant papers (10%) at the full‐text level (JC). Final decisions on study inclusion/exclusion were made by consensus of all three authors (EG, JC and AJW).

**TABLE 2:** Inclusion and exclusion criteria used to select included studies for this systematic review.

	Inclusion	Exclusion
Population	Participants‐ 8–18 years	< 8 years and > 18 years
		Participants with known underlying health conditions (except overweight/obesity)
Intervention/study design	Quantitative analysis present: observational, prospective, longitudinal, cohort, cross‐sectional, nested, intervention, trial, clinical study and randomised‐controlled trials.	Qualitative reviews, descriptive reviews, articles, case reports, letters, abstracts only, unpublished work, and summary studies.
Comparison	Weight status subgroups or sleep health parameter subgroups if intervention allows.	
Outcomes	Sleep measured as an outcome or an exposure using validated quantitative or qualitative (objective and subjective) methods, including but not limited to polysomnography, actigraphy, sleep diaries, sleep questionnaires or a combination of methods. Sleep measures included but were not limited to sleep quality, efficiency, fragmentation, onset latency, time in bed, and insomnia.	No clear information on how sleep measurements have been taken or caregiver‐reported sleep only.
	Obesity or Adiposity measured objectively. Obesity and adiposity measures included but were not limited to BMI z‐scores, BMIp, waist circumference, hip circumference, skinfolds and body fat percentage.	Self‐reported obesity measurements
	Circadian misalignment or chronotype is measured subjectively or objectively.	No clear information on how circadian misalignment or chronotype has been measured.
		Studies solely investigating sleep duration.

Abbreviations: BMI – body mass index; BMIp – body mass index percentile.

### 2.3 Inclusion and Exclusion Criteria

The PICOS framework (Richardson et al. 1995) was used to identify included studies (Table 2). Eligible participants were 8–18 years to account for adolescence and for the changes at the start of puberty, which can start around age 8 years in girls (Llop‐Viñolas et al. 2004). Inclusion criteria also included full‐text availability, peer‐reviewed studies, English language only and human studies only (Table 2). Eligible studies were those that investigated the relationship between the determinant and both sleep and obesity to ensure that determinants were measured in the same way.

### 2.4 Quality Assessment of the Studies

Study quality was assessed using two JBI tools—one for cross‐sectional studies (max score out of 8) and one for cohort studies (this included the intervention, cohort and longitudinal studies) (max score out of 11) (Moola et al. 2020). Both tools were pre‐selected based on the study designs in the inclusion criteria (Table 2). Scores allocated for each criterion were awarded 1 point for “yes” no points for “unsure” “not applicable” or “no” Scores were then converted to percentages to make them comparable across study designs. A quality assessment score of 75% and above was used to categorise a higher‐quality study, and those below 75% were categorised as a lower‐quality study. A calibration exercise was conducted with 10% of included papers by three authors (EG, AJW and JC) before the remaining 90% of papers were quality assessed by the first author (EG).

## 3 Results

### 3.1 Data Extraction Recording and Findings

**FIGURE 1:**
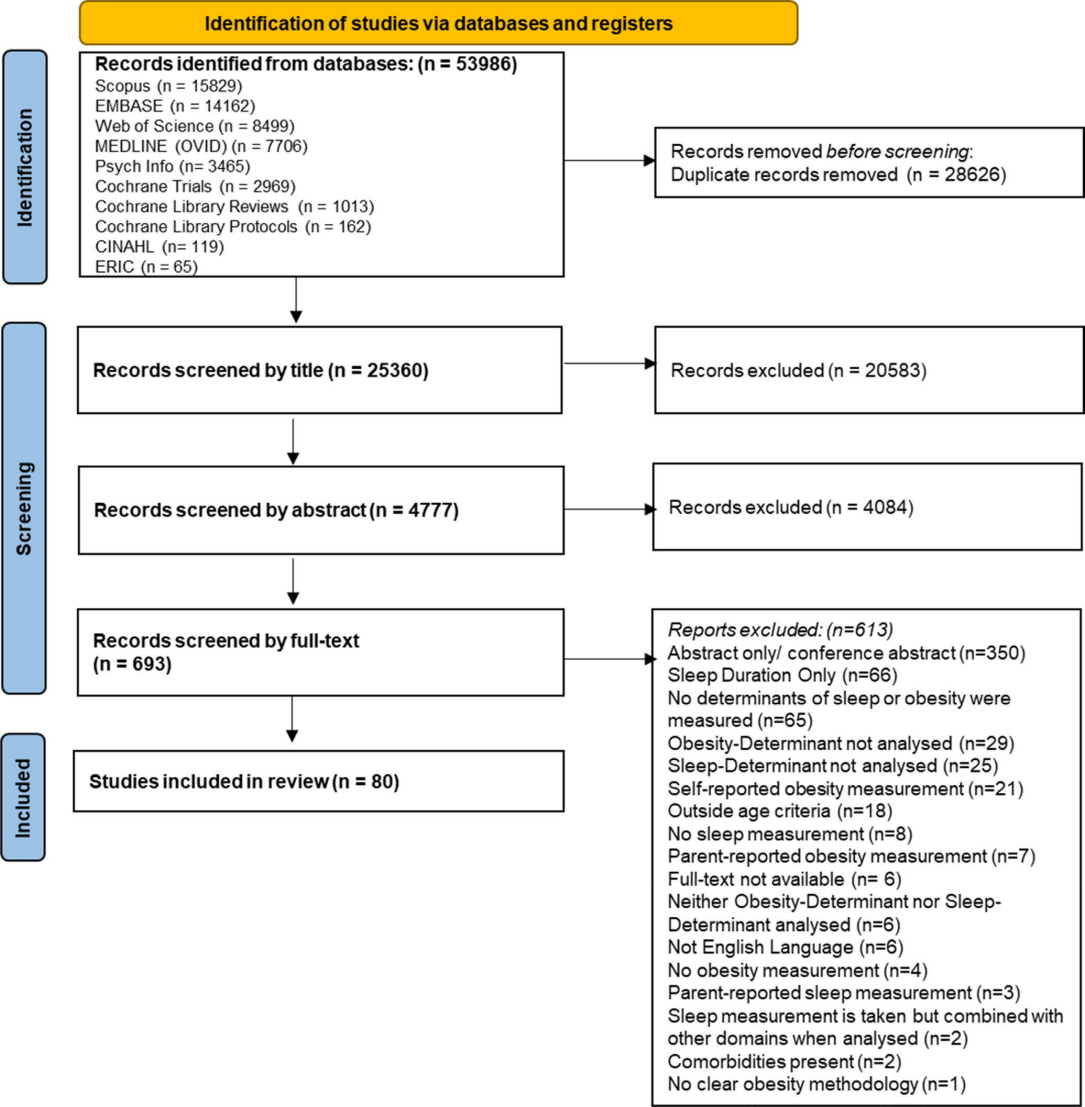
PRISMA Diagram showing the number of studies included and excluded at each screening stage (Page et al. 2021).

The initial search identified 53,986 records matching the search terms across the 10 databases (Figure 1). Of the 693 records identified for screening at the full‐text level, 350 were conference abstracts or abstracts only (the full body of text was not present) and 6 were behind inaccessible paywalls. Subsequently, 337 records were accessed and screened at the full‐text level. Overall, 80 records met the inclusion criteria for this systematic review (Table 3).

**TABLE 3:** Associations between the determinant and sleep, as well as obesity or adiposity measures.

Author (year) *country*	Study design	Participants			Outcomes and variables			Results				Quality (%)
		Sample size	Mean age (Range) (years)	Male (%)	Determinant variable	Adiposity outcome	Sleep outcome	Analysis	Obesity‐determinant	Sleep‐determinant	Adjusted variables	
Adelantado‐Renau et al. (2019) *Spain*	CS	262	13.9 ± 0.3	52.3	Academic ability, gender	BMI, WS	SQ	Regression	WS: Maths—NEG*; Language—NEG*; GPA—NEG*	Maths POS*; Language POS*; GPA POS*	2, 5, 6	75
								T‐test	BMI: Gender—NS	Gender—Boys***	1	
Bagley and El‐Sheikh (2013) *USA*	CS	228	10.4 (9.1–12.3)	54.4	CRI	BMIz	SE	Regression	POS**	NS	2, 3, 4, 5, 6	75
								Predictor modelling	SE→BMIz mediated by cumulative risk index**. Three levels of risk: Step 1—Controls ** Step 2—Sleep and Cumulative Risk ** Step 3—Sleep X Cumulative Risk**			
Bagley and El‐Sheikh (2014) *USA*	CS	235	11.3 ± 0.6	52.3	PEP‐R	BMIz	SE; Sleep activity; Long wake periods	Correlation	NS	NS	1	75
								Predictor modelling	SE→PEP reactivity**; Sleep activity→PEP reactivity**; Long wake periods→PEP reactivity**		2, 33, 34	
Bagley et al. (2015) *USA*	Long	256	10.4 ± 0.7	54.3	CRI (T1)	BMIz (T1), BMIz (T2)	SE (T1), SOvar (T1)	Regression	BMI (T1): POS** BMI (T2): POS**	NS	2, 3, 4, 6, 10	90.9
								Predictor modelling	Variability of SO T1→Cumulative Risk Index→BMI (T2)**			
Bates et al. (2016) *USA*	INT	60	11.8 ± 1.0	0	MVPA (T1), MVPA (T2)	BMIz (T1; T2), BMIp (T1; T2)	SO (T1;T2), WT (T1;T2)	Correlation	NS	SO (T1); WT (T2): NS SO (T2): MVPA (T1)—NEG* WT (T1): MVPA (T2)—NEG*	1	54.4
Bottolfs et al. (2020) *Norway*	CS	611	13.2	50.9	Gender, Health quality	BMI	SDis	Correlation	Gender—Girls**	Gender—Girls**	1	87.5
								Regression	Physical wellbeing—NEG* Psychological wellbeing; caregiver relations and autonomy, social and peer support; school environment—NS	Physical wellbeing; psychological wellbeing; caregiver relations and autonomy; school environment—NEG* Social and peer support—NS	1	
									Physical wellbeing—NEG*	Physical wellbeing; psychological wellbeing; caregiver relations and autonomy; school environment—NEG*	2, 5, 11	
Cardoso et al. (2024) *Portugal*	CS	560	10.0 ± 2.7	47.6	Healthy Eating	WS	Chronotype	Regression	NEG**	NEG**	2, 3, 13	87.5
Catalan‐Lamban et al. (2023) *Spain*	INT	122	Control: 10.7 ± 2.3 Int: 11.5 ± 2.5 (2 year. follow‐up)	62.3	Diet education intervention	BMIz, BF%, WC	SOL, SE, TIB, TST, WASO, number of awak, dur of awak	T‐test	Int and control group—BMI NEG** Int compared with control group—BF% NEG**, WC NEG**	Int group compared with baseline— SE POS*** SOL, WASO, awak dur NEG***	1	81.8
Chaput et al. (2014) *Canada*	CS	502	10.0 ± 0.4	41	Screentime (number and type of screens)	BF%, WS	SE	Regression	BF%: Number of screen—POS*; TV screen—POS*; Computer screen—NS WS: Number of screen—POS*	NEG**	2, 3, 4, 12, 13	87.5
Chaput et al. (2023) USA	CS	996	8.3 ± 1.2	ND	Gender	BMIz	SE, sleep regularity	T‐test	Girls POS***	NS	1	87.5
da Silveira et al. (2018) *Brazil*	CS	245	12	44.5	DMFT	BMI	Chronotype	Correlation	NS	NS	1	75
Dema et al. (2019) *Bhutan*	CS	5809	(13.0–17.0)	46.2	Suicide Ideation	WS	SO Anxiety	Correlation	POS**	POS***	1	62.5
Dong et al. (2023) *USA*	CS	142	14.0 ± 1.4	41.0	Food insecurity (SES)	BMIz, WC	Sdis, Sleep health	Regression	BMIz—POS*	Sdis‐POS***	2, 3, 31	87.5
								Path analysis	Indirect: Food insecurity→Lower sleep health→Higher WC*			
Dos Santos et al. (2024) *Brazil*	CS	1010	13.2 ± 2.4	45.0	Physical activity	BMI	SQ	T‐test	NS	NS	1	87.5
								Regression	In those with insufficient physical activity, higher screen use (adjusted for BMI), was significantly associated with poorer sleep quality*		2, 3, 33	
Ekstedt et al. (2013) *Sweden*	CS	1231	(6.0–10.0)	49.6	MVPA, Sed time	BMI, WS	SE, SO, WT	Correlation	BMI: MVPA—NEG**; Sed time—NS WS: MVPA—NEG**; Sed time—NS	SE: MVPA—POS**; Sed time—NS SO: NS WT: MVPA—POS**; Sed time—NS	2, 3	62.5
								Path analysis		MVPA**→**SE** Sed time**→**SE** SO**→**MVPA** MVPA**→**SO** SO**→**Sed time** Sed time**→**SO** WT**→**MVPA** MVPA**→**WT** WT**→**Sed time** Sed time**→**WT**	2, 3	
El‐Sheikh et al. (2007) *USA*	CS	167	8.7 ± 0.3	45	Int problems, Ext problems, Depression, Vagal time, Vagal regulation	BMI	SE	Regression	Vagal tone—NEG** Int problems; Ext problems; Depression; Vagal regulation—NS	Int problems—NEG*** Ext problems—NEG** Depression; Vagal tone; Vagal regulation—NS	2, 3, 4, 5	87.5
El‐Sheikh et al. (2019) *USA*	CS	235	15.8 ± 0.8	46	Int problems, Aggressive behaviour, Rule breaking behaviour	BMIz	SQ	Correlation	NS	NEG***	1	75
								Predictor modelling	Int problems—POS** Aggressive behaviour; Rule breaking behaviour—NS	NEG***	2, 3, 4, 5, 14	
Eskenazi et al. (2019), *USA*	CS	397	16	47.8	Gender, Immigration vulnerability	BMIp	SQ	T‐test	Gender—Girls**	Gender—Boys*	1	87.5
								Regression	Immigration vulnerability—NS	Immigration vulnerability—Threat to family—NEG*, children vulnerability—NEG*	2, 12, 13, 15, 16, 17	
Ferranti et al. (2016) *Italy*	CS	1586	12 ± 0.7	54.9	Diet (Med)	BMI	Sleep timing, SQ	Correlation	NEG**	NS	1	87.5
Ferrari et al. (2017) *Brazil*	CS	328	10.4 ± 0.5	51.5	Gender	BF%, WC	SQ	Regression	Girls***	NS	2, 4, 18	87.5
Ferrari et al. (2019) *Brazil*	CS	328	10.4 ± 0.5	51.5	Gender	BF%, BMI, WC, WS	SQ	Regression	BF%; WC—Girls***; WS—Girls*; BMI—NS	NS	2, 4, 18	87.5
Fujimura et al. (2019) *Japan*	CS	9492	12.3 ± 0.4	50.1	Gender	BMI, WS	SQ	T‐test	BMI—Girls**; WS—NS	Boys***	1	75
Gan et al. (2019) *Malaysia*	CS	421	13.3 ± 1.3	41.8	SSB	BMIz	SQ	Correlation	POS*	NEG***	1	62.5
García‐Hermoso et al. (2017) *Chile*	CS	395	12.1 ± 0.7	50.3	Gender	BMI, BW, WC, WS	BT refusal, Sleep habits Sleep routine, SQ, SO Anxiety	T‐test	NS	SO Anxiety; BT refusal—Girls***; Sleep habits and sleep routine—Boys***; SQ—NS	1	87.5
								Partial coefficient correlation	SQ – BMI; WC NS SO Anxiety—BMI*; WC* BT refusal‐ BMI***; WC—NS Sleep Routines – WC*; BMI—NS Sleep Habits—BMI*; WC*		3	
Golley et al. (2013) *Australia*	CS	2200	12.9 ± 2.2 (9.0–16.0)	49	Gender	BMIz, WS	Sleep timing	T‐test	NS	NS	1	87.5
Goodman et al. (2020) *UK*	Long	16,376	T1–5.0; T2–7.0; T3–11.0; T4‐14.0	51.3	Videogaming	BMIz	BTVar	Correlation	POS*	POS*	1	81.8
Gupta et al. (2002) *USA*	CS	361	13.0 ± 1.7 (11.0–16.0)	46.2	Pubertal status, ethnicity, drug use, alcohol use, tobacco use, physical activity	WS	Sdis	Correlation	Pubertal status—POS* Ethnic minorities—POS* Drug use; Tobacco use; Alcohol use; Physical activity—NS	Physical activity—NEG* Pubertal status; Ethnicity, Drug use; Tobacco Use; Alcohol use—NS	1	62.5
Harrex et al. (2018) *New Zealand*	CS	439	10.2 ± 0.6	49	Gender	BMIz, WS	Sleep timing, SO, WT	T‐test	NS	WT—Girls** SO; Sleep timing—WS	1	62.5
Harrington et al. (2021) *UK*	CS	816	12.8 ± 0.8	0	Screentime (Number of screens)	BMI	TIB	Correlation	POS*	NEG*	1	75
He et al. (2015) *USA*	CS	324	16.7 ± 2.3	51.9	Ethnicity	BMIp	SleepVar	Correlation	Non‐white Hispanic v Ethnic Minority ***	Non‐white Hispanic v Ethnic Minority *	1	75
He et al. (2020) *USA*	CS	324	16.7 ± 2.3	51.9	Ethnicity	BMIp	SleepVar	Regression	Non‐white Hispanic v Ethnic Minority ***	Non‐white Hispanic v Ethnic Minority *	2, 3, 6, 27, 36, 37	75
Herttrich et al. (2020) *Germany*	CS	151	Prepubertal 7.2 ± 1.0 Pubertal 13.4 ± 1.1	47	Pubertal status	BF%, BMI, BMIz, HC, Skinfolds, WC, W:Hi	AHI, Arousal index	Correlation	POS***	Arousal index—POS* AHI—NS	1	75
Hjorth, Chaput, Damsgaard et al. (2014) *Denmark*	CS	723	10.0 ± 0.6	52	Gender	BMIz, FMI, WC, WS	Sleep habits	T‐test	FMI—Girls*** BMIz; WC; WS—NS	NS	1	75
Hjorth, Chaput, Gao et al. (2014) *Denmark*	CS	785	10.0 ± 0.6	52	Gender	BF%, BMIz, FMI, FFMI, WC	Sleep habits	T‐test	BF%; FFMI; FMI—Girls*** BMIz; WS—NS	NS	1	75
								Combined Cross‐sectional Associations	Sleep Habits and Sed Time→FMI * Sleep Habits and Screen time→FMI * Sleep Habits and MVPA→FMI *** Sleep Habits, Sed Time, Screen time→FMI * Sleep Habits, MVPA, Screen time→FMI * Sleep Habits, MVPA and Sed Time→FMI ***			
Hjorth et al. (2016) *Denmark*	CS	790	9.9 ± 0.6	51	Academic attainment	WS	Sleep habits	Regression	D2 Test‐Concentration performance and error percentage—NEG* Maths; Reading speed; Reading accuracy—NS	Maths—POS*** Reading accuracy—POS* D2 Test‐Concentration performance and error percentage; reading speed—NS	2, 3, 4, 7, 8, 9, 13, 18, 19, 20, 21	87.5
									Reading speed; Reading accuracy—NEG* D2 Test‐Concentration performance and error percentage; maths—NS	Maths—POS* D2 Test‐Concentration performance and error percentage; reading speed and accuracy—NS	2, 3, 4, 7, 8, 9, 13, 14, 18, 19, 20, 21, 22, 23, 24, 25	
Hrafnkelsdottir et al. (2020) *Iceland*	CS	247	15.8 ± 0.3	41.7	Gender	BF%, BMI	BT, BTVar, Number of Awak Var, SE, SEVar, SOL, SOLVar, Sleep timing, TIB, TIBVar, WT, WTVar	T‐test	BF%: Girls *** BMI: NS	BT; BTVar; TIBVar—Girls** WT; Number of awak Var—Girls*** WTVar—Girls* SEVar—Boys* Number of Awak; SE; SOL; SOLVar; Sleep timing; TIB—NS	1	75
Iglayreger et al. (2014) *USA*	CS	37	14.0 (11.0–17.0)	54.1	Gender	BF%, BMI, BMIz, WC	Napping	T‐test	BMIz: Girls* BF%; BMI; WC—NS	NS	1	87.5
Jankovic et al. (2023) *Germany*	CS	81	(9–16)	59.0	Diet Chrono Alignment (1‐year follow‐up)	BMIz, FFMI	Chronotype	Regression	BMIz—NS FFMI higher in later chronotype that eat later** ∆ FFMI higher in later chronotypes that eat later*		2, 3, 6, 25, 32, 37, 43, 44, 45	100.0
John et al. (2023) *India*	CS	184	14.9 ± 1.0	64.7	Screentime	BMI	BT, WT	Chi^2^	NS	BT WD POS***, BT WE POS**, WT WD NS, WT WE POS*	1	37.5
Kathrotia et al. (2010) *India*	CS	142	17.8 ± 0.8	73.2	Gender	BMI	BTVar, Daytime sleepiness, SO, SOL, WT, WTVar	T‐test	NS	NS	1	75
Kracht et al. (2019) *USA*	CS	256	12.4 ± 1.9	45.3	Diet (Energy intake, Food cravings, Healthy eating)	BMIz	SE	Correlation	NS	Energy intake; Food cravings—NEG* Healthy eating—NS	1	87.5
LaVoy et al. (2020) *USA*	CS	55	12.2 ± 2.0	47	Gender	BMIz	SE; Sfrag; Sleep habits; SOL; WASO	T‐test	NS	Sfrag—Girls*; Sleep habits—Boys*; SE; SOL; WASO—NS	1	75
LeMay‐Russell et al. (2021) *USA*	Long	137	12.5 ± 2.6	46	Depressive symptoms, Ethnicity, Gender, Pubertal status, Season, SES	BMIz, FM (T1; T2)	BT; BTVar; SJ; SleepVar; Sleep Mid‐point; WT; WTVar	Correlation	BMIz: Ethnicity—AA/NHB**; SES—NEG*; Depressive symptoms; Gender; Season; Pubertal status—NS FM (T1): Ethnicity—AA/NHB*; Gender—Girls**; Pubertal status—POS**; SES; Depressive symptoms; Season‐ NS FM (T2): Gender—Girls**; Pubertal status—POS**; SES; Gender; Depressive symptoms; Season‐ NS	BT: Ethnicity—AA/NHB**; Pubertal status—POS**; Depressive symptoms; Gender; Season; SES—NS BTVar: SES—NEG**; Ethnicity; Gender; Depressive symptoms; Pubertal status; Season—NS SJ: Pubertal status—POS*; SES—NEG**; Winter* Ethnicity; Gender; Depressive symptoms—NS SleepVar: Ethnicity—AA/NHB**; Pubertal status—POS*; Depressive symptoms; Gender; Season; SES—NS Sleep Mid‐point: Ethnicity—AA/NHB**; Pubertal status—POS**; Depressive symptoms; Gender; Season; SES—NS WT: Ethnicity—AA/NHB**; Pubertal status—POS*; Winter*; Depressive symptoms; Gender; Season; SES—NS WTVar: Ethnicity—AA/NHB**; Pubertal status—POS*; Winter*; Depressive symptoms; Gender; Season; SES—NS	1	63.6
Lemola et al. (2011) *Finland*	CS	291	8.1 ± 0.3	48.5	Gender	BMI	SE, SOL	T‐test	NS	SE: Boys** SOL: NS	1	87.5
Lima et al. (2020) *Brazil*	CS	1242	15.1 ± 0.9	44.4	Anxiety, Depressive symptoms, Gender, Maternal education level	Skinfolds	SQ	Correlation	Gender—Girls***	Gender—Boys***	1	75
								Path analysis	Anxiety→Skinfolds * Anxiety→SQ * SQ→Depressive symptoms* Depressive symptoms→Skinfolds* Anxiety➔ SQ➔ Depressive Symptoms➔Skinfolds* Maternal education level – NS		2, 3, 13, 26	
Lin et al. (2020) *Iran*	Long	861	15.9 ± 3.2	43.2	Eating disorder attitudes, Food addiction, Psychological distress, Gender, Caregiver education level and Caregiver BMI	BMIz (T1;T2)	Insomnia symptoms (T1;T2)	Mediation analysis	Eating disorder attitudes→BMIz*** Food addiction→BMIz*** Psychological distress→Insomnia symptoms→BMIz*** Psychological distress→Food addiction→BMIz *** Psychological distress→Eating disorder attitudes→BMIz *** Psychological distress→Insomnia***		1	90.9
								Correlation	Eating disorder attitudes; Food addiction; Psychological distress; Maternal BMI—BMIz (T1;T2)—POS** Father's BMI—BMIz (T2) POS*, BMI (T1)—NS Father's education level—BMIz(T2)—NEG* Gender‐NS	Eating disorder attitudes; Food addiction; Psychological distress—Insomnia symptoms (T1;T2)—POS** Gender—Girls** Father's education level—Insomnia symptoms (T2)—NEG* Maternal and father BMI—NS		
								Path analysis	Eating disorder attitudes→BMIz** Food addiction→BMIz** Psychological distress→Insomnia symptoms→BMIz*** Psychological distress→Food addiction→BMIz *** Psychological distress→Eating disorder attitudes→BMIz *** Psychological distress→Insomnia***			
Lucas‐de la Cruz et al. (2018) *Spain*	CS	146	9.4 ± 0.74	45.2	Gender	BF%, BMI, BW, WC	BT, SE, SOL, TIB, WT	T‐test	BF%—Girls** BMI; BW; WC—NS	SE—Boys*; TIB—Girls* BT;WT;SOL—NS	1	87.5
Lytle et al. (2011) *USA*	CS	723	14.7 ± 1.8	48.8	Gender	BF%, BMIp, WS	BTVar, SJ, Night eating, WT	T‐test	BF%—Girls*** BMIp; WS—NS	BT Var; SJ—Girls* Night eating; WT—NS	1	87.5
Magalhaes et al. (2020) *Brazil*	CS	212	15.6 ± 1.9	26.9	Gender	BF%, BMIs, BW, WC	SQ	T‐test	NS	NS	1	62.5
Moitra et al. (2020) *India*	CS	527	(10.0–17.0)	42	Depressive symptoms	WS	Daytime sleepiness, Insomnia symptoms	Correlation	POS*	Daytime sleepiness; Insomnia symptoms—NS	1	75
								Regression	NS	Insomnia symptoms—POS***; Daytime sleepiness—NS	2, 3	
Moitra et al. (2021) India	CS	772	13.2 ± 1.4 (10.0–15.0)	51	Gender	WC, W:He, WS	SQ	T‐test	W:He; WC—Girls* WS—NS	Boys*	1	87.5
Moore et al., (2007a) *USA*	CS	247	13.6 ± 0.7	51.4	Anxiety, Depressive symptoms, Perceived health	BMIp	Daytime sleepiness	Predictor modelling	NS	Daytime sleepiness→Anxiety*** Daytime sleepiness→Depressive symptoms*** Daytime sleepiness→Percieved health***	2, 3, 4, 6, 12, 13, 27, 28, 29	75
Morrissey et al. (2019) *Australia*	CS	2253	10.9 ± 1.1	50.2	SSB (consumption; 1‐h before bed), Take away consumption, Snacking, Fruit and vegetable consumption, Screentime (in bed; 1‐h before bed; guidelines), Physical activity (1‐h before bed; guidelines)	WS	Sleep health	Correlation	SSB (consumption—POS*; 1‐h before bed—NS), Take away consumption—POS**, Snacking—NS, Fruit and vegetable consumption—NEG*, **Screentime** (in bed—NS; 1‐h before bed—POS**; guidelines—NEG*), Physical activity (1‐h before bed—NEG*; guidelines—NEG*)	SSB (consumption—NEG**; 1‐h before bed—NEG***), Take away consumption—NEG*, Snacking—NEG*, Fruit and vegetable consumption—NS, Screentime (in bed—NEG***; 1‐h before bed—NEG***; guidelines—POS*), Physical activity (1‐h before bed—NEG***; guidelines—POS***)	1	75
Negele et al. (2020) *Germany*	CS	1223	15.6	45	Gender	BMI	SE, SOL, WASO	T‐test	Girls*	SE—Boys*; SOL—Girls*; WASO—Girls*	1	75
Ogutlu et al. (2023) *Turkey*	CS	35	(10‐17)	28.6	Cognitive function (sluggish cognitive tempo and cognitive problems/inattention)	BMI	Daytime sleepiness	Correlation	NS	Sluggish cognitive temp POS***, Cognitive problems/inattention POS**	1	62.5
Olds et al. (2011) *Australia*	CS	2200	(9.0–16.0)	49.4	Gender	WS	BT	T‐test	NS	NS	1	62.5
Ozkan et al. (2020) *Turkey*	CS	346	11.9 ± 0.8	50.9	Gender	HC, WS	SQ	T‐test	NS	NS	1	87.5
								Correlation, separated by gender	**HC:** Boys Good SQ v Bad SQ‐ NS; Girls Good SQ v Bad SQ *			
Pabst et al. (2009) *USA*	CS	264	14.9 ± 2.2	0	Depressive symptoms	WS	Chronotype	Regression	NS	Later POS***	3, 4, 5, 6	87.5
								Correlation separated by WS	OW/OB evening preference and depressive symptoms*			
Panagiotou et al. (2023) *Greece*	INT	32	8.0 ± 0.2	46.9	PSAI‐CA (stress and QOL management and lifestyle education (healthy nutrition, sleep, physical activity, bullying, and screens (mobile‐tablet‐PC) intervention (8‐weeks)	BMIz	BT	T‐test	Int compared with baseline: NEG**	Int compared with baseline: NS	1	63.6
Parker et al. (2022) *USA*	CS	48	12.9 ± 2.7	31.2	Eating behaviour—loss of control, Food cravings	FM, Height	Sleep mid‐point, SO, WT	Correlation	NS	Sleep mid‐point: eating behaviour POS**; food craving—NS SO: NS WT: eating behaviour POS**; food craving—NS	2, 3, 4, 5, 16, 39, 40, 41	100
Pickett et al. (2022) *USA*	CS	181	14.3 ± 1.5	32.6	Food addiction	WS	Daytime sleepiness	Correlation	POS*	POS***	2, 3	100
Pompeia et al. (2023) *Brazil*	CS	278	(9‐15)	39.6	Gender	BMI, BF%, W:He	SO, WE, SJ	T‐test	BF% Girls***; BMI NS	SJ Boys**; SO, WE—NS	1	75
Quante, Khandpur et al. (2019) *USA*	CS	669	12.9 ± 0.6	49	Season	BMI, BMIz	SE, Sleep mid‐point, WASO	Regression	BMI—Winter*; BMIz—Winter*	SE—Summer***, Sleep mid‐point—Winter***, WASO—Winter***	2, 3, 4, 6, 12, 13, 30	87.5
Roberto et al. (2023) *Brazil*	CS	1333	(7‐14)	43.3	Gender	WS	BT, WT, Mid sleep point	T‐test	Boys***	BT—NS WT WE—Girls***	1	87.5
					Snack timing	WS	Chronotype	Regression	Higher WS—less breakfast and more evening snacks***	Early chronotype—morning snack*** Late chronotype—evening snack ***	2, 3, 13, 18, 25, 37, 38, 46, 47	
Rognvaldsdottir et al. (2020) *Iceland*	CS	252	15.8 ± 0.3	42	Gender	BF%, BMI, BW, WC, TF%	BT, BTVar, SleepVar, TIB, WASO, WT	T‐test	BF%; BW; WC; TF%—Girls*** BMI—NS	NS	1	87.5
Rosi et al. (2017) *Italy*	CS	690	10.8 ± 0.4	48.3	Diet (Med)	WS	Sleep timing	Correlation	NS	NS	1	32.5
Rosli et al. (2021) *Malaysia*	CS	85	(9.0–12.0)	54.1	Cognitive function	WS	Daytime sleepiness	Correlation	NS	NS	1	62.5
Saleh‐Ghadimi et al. (2019) *Iran*	CS	150	15.6 ± 1.7	0	Emotional eating, Energy intake	BMI	SQ	Path analysis	BMI→Carbohydrate intake* BMI→Emotional eating—NS BMI→Energy intake—NS BMI→Fat intake—NS BMI→Protein intake—NS	SQ→Emotional eating* SQ→Energy intake* SQ→Fat intake* SQ→Carbohydrate intake—NS SQ→Protein intake—NS	1	62.5
Shakir et al. (2018) *Australia*	CS	234	11.9 ± 1.2	56		BF%, W:He	BT, WT	T‐test	BF% Girls**; W:He—NS	NS	1	87.5
Skjakodegard et al. (2021) *Norway*	Cohort	170	12.1 ± 2.8	41.2	MVPA, Screentime	WS	Sleep mid‐point, SJ	Regression	MVPA***; Screentime—NS	Sleep mid‐point: MVPA—NEG*; Screentime—POS* SJ: MVPA—NEG*; Screentime—NS	2, 3, 13, 31	63.6
Snell et al. (2007) *USA*	Long	1667	T1: 8.1 ± 2.9; T2: 13.7 ± 2.9	50	Caregiver education level, Ethnicity, SES	BMI (T1;T2)	BT (T1); WT (T1)	Correlation	BMI (T1): Ethnicity—AA*, Non‐Hispanic White** and Hispanic*; SES—NEG*; Caregiver education level—NEG** BMI (T2): Ethnicity—Hispanic*, Non‐white Hispanic and AA—NS; SES—NS; Caregiver education level—NS	BT: Ethnicity—AA**, Non‐White Hispanic**, Hispanic—NS WT: NS	1	90.9
Stoner et al. (2018) *New Zealand*	CS	341	9.6	50	Gender	BF%, BMI, BW, FM, FMI, WS, W:Hi	BT, SJ, WT		BF%—Girls***, FM—Girls**, FMI—Girls*, W:Hi—Girls* BMI, BW, WS—NS	SJ: Girls*; BT: WD and WE—NS; WT: WD—NS, WE—Girls*	1	87.5
Tabatabaee et al. (2018) *Iran*	CS	904	15.0 ± 1.3	49	Fast food consumption, internet addiction, physical activity	BMIp	SQ	Path analysis	Internet addiction→BMIp*** Internet addiction→SQ*** Internet addiction→SQ→Fast food consumption→BMIp*** Internet addiction→SQ→Fast food consumption*** Internet addiction→SQ→BMIp*** Internet addiction→Physical activity→BMIp*** Internet addiction→SQ→Physical activity***		1	62.5
Tee et al. (2018) *Malaysia*	CS	513	14.1 ± 1.3	41.1	Cognitive flexibility, neurocognitive inhibition, working memory	BMIz	SE, SQ	Correlation	Neurocognitive inhibition—POS*, Working memory—NEG*, Cognitive flexibility—NS	**SE:** Cognitive flexibility—NS, neurocognitive inhibition—POS**, working memory—POS** **SQ:**	1	87.5
Wang, Adab et al. (2017) *China*	CS	5518	10.2 ± 0.9	53.9	Gender	BF%, BMI, BMIz, WC, W:He, WS	BT, SDis	T‐test	Girls***	NS	1	87.5
Wang et al. (2024) *China*	INT	136	(9–12) PEG: 11.0 ± 0.4 ISMG: 11.1 ± 0.7 Control: 11.0 ± 0.6	ND	Exercise and medical education intervention (ISMG—intergration of sports and medicine; PEG—physical exercise only).	BMI	SQ	ANOVA and T‐tests	PEG and ISMG intervention groups compared with control and compared with baseline: BMI NEG** **No difference between intervention groups**	PEG and ISMG intervention groups compared with control and compared with baseline: SQ POS** **No difference between the intervention groups**	1	63.6
Werneck et al. (2018) *Brazil*	CS	280	14.7 ± 2.0	68.6	Gender	BF%, BMI	SQ	T‐test	BF%—Girls***; BMI—NS	NS	1	87.5
Winpenny et al. (2023) *UK*	CS	815	14.5 ± 0.3	43.5	Gender	BMIz	Sleep mid‐point	T‐test	NS	Girls*	1	87.5
Yaghtin et al. (2022) *Iran*	CS	1026	(12.0–18.0)	0	Diet (med)	BMIp, WC	Insomnia symptoms	Correlation	BMIp—NEG*; WC—NS	NEG*	1	87.5
								Regression		NEG*	37	
										NEG*	6, 13, 25, 37	
										NEG*	6, 13, 25, 27 37, 42	
Zhang et al. (2024) USA	Long	3326	(10.6–13.7)	50.7	Gender (T1), Ethnicity (T1), Parental highest education (T1), Puberty status (T1), SES (T1), Externalising problems (T5; T7), Internalising problems (T5; T7)	BMI (T1)	SE (T5), SOL (T5), WASO (T5), Midpoint (T5)	Correlations	Gender—NS Ethnicity (T1)** Parental highest education (T1)**, Puberty status (T1)**, SES (T1), Externalising problems (T5**; T7**), Internalising problems (T5**; T7**)	**SE (T5):** Gender—boys*, Ethnicity (T1)** Parental highest education (T1)*, Puberty status (T1)‐NS, SES (T1)‐NS, Externalising problems (T5*; T7—NS), Internalising problems (T5*; T7—NS) **SOL (T5):** Gender—boys*, Ethnicity (T1)* Parental highest education (T1)‐NS, Puberty status (T1)*, SES (T1)‐NS, Externalising problems (T5; T7—NS), Internalising problems (T5; T7—NS) **WASO (T5):** Gender—boys**, Ethnicity (T1)** Parental highest education (T1)**, Puberty status (T1)**, SES (T1)**, Externalising problems (T5**; T7**), Internalising problems (T5**; T7**) **Sleep mid‐point (T5):** Gender—boys**, Ethnicity (T1)** Parental highest education (T1)**, Puberty status (T1)**, SES (T1)**, Externalising problems (T5**; T7**), Internalising problems (T5**; T7**)	1	90.9

*Note*: Significance: POS‐ Significant Positive Correlation; NEG‐ Significant Negative Correlation; NS‐ Not Significant; ***p < 0.001; **p < 0.01; *p < 0.05. Timings: T1—Timepoint 1; T2—Timepoint 2. Adiposity Measures: BF%, Body Fat Percentage; FFMI, Fat‐Free Mass Index; FMI, Fat Mass Index; FM, Fat Mass; HC, Hip Circumference; TF%, Trunk Fat Percentage; WC, Waist Circumference; W:He, Waist to Height Ratio; W:Hi, Waist to Hip Ratio. Obesity Measures: BMI, Body Mass Index; BMIz, Body Mass Index Z‐scores; BMIp, Body Mass Index Percentiles; BW, Body Weight; HW, Healthy Weight; OB, Obese; OW, Overweight; WS, Weight Status. Sleep Outcomes: BT, Bedtime; SDis, Sleep Disturbance; SE, Sleep Efficiency; SFrag, Sleep Fragmentation; SO, Sleep Onset; SOL, Sleep Onset Latency; SQ, Sleep Quality; SOL, Sleep Onset latency; TIB, Time in Bed; WASO, Wake After Sleep Onset; WD, Weekday; WE, Weekend; WT, Wake Time. Determinants: AA, African American; CRI, Cumulative Risk Index; F, Female; KIDMED, Mediterranean Diet Quality Index in Children; M, Male; MVPA, Moderate‐Vigorous Physical Activity; NHB, Non‐Hispanic Black; PEP, Pre‐ejection Period. Study design: CS, Cross‐sectional; Int, Intervention; Long, Longitudinal; RC, Retrospective Cohort. Additional abbreviations: ND, Not defined. Adjusted Variables: 1—Unadjusted; 2—Gender/Sex; 3—Age; 4—Ethnicity/Minority Status; 5—Socioeconomic Status; 6—Pubertal Status; 7‐Year Group at School; 8—Siblings; 9‐Class Type; 10—Asthma; 11—Place of Birth; 12—Household Income; 13—Caregivers Education; 14—Sleep Habits; 15—Marital Status; 16—Depressive Symptoms; 17—Length of Time since Emigration; 18—School Type; 19—Month of Measurement; 20—Time of Measurement; 21—Day of Measurement; 22—Breakfast Consumption; 23—Cardiorespiratory Fitness; 24—Sedentary Time; 25—Physical activity; 26—Caregivers Height; 27—BMIp; 28—Preterm Status; 29—Vacation Status; 30‐BMI z‐scores; 31—Caregivers Cohabitation; 32—Season of Measurement; 33—BMI; 34—Pre‐ejection Period; 35—Caregivers BMI; 36—Sleep Variability; 37—Energy Intake; 38—Weight Status; 39—Fat Mass; 40—Height; 41—Snoring; 42—Waist circumference; 43—Number of questionnaires; 44—Smoking in the household; 45—Social jetlag; 46—Screentime; 47—Sleep Duration.

### 3.2 Study Characteristics

The studies were completed in 23 countries (across 6 continents) and consisted of 69 cross‐sectional studies, 6 longitudinal studies (repeated measures), 4 intervention studies and 1 cohort study. Thirty‐eight sleep measures, 15 adiposity and obesity measures, and 79 determinant measures were examined across the 80 studies in the systematic review (Table 3). The mean quality appraisal percentage of included studies was 78.4% ± 12.7% (Tables 4 and 5). The studies used a range of analyses including descriptive statistics, unadjusted inferential statistics and more complex inferential statistics such as path analysis, mediation analysis, partial correlation coefficients, and regression analysis.

**TABLE 4:** Quality appraisal of all included cross‐sectional studies using the JBI cross‐sectional studies tool.

JBI study quality—Cross‐sectional studies											Quality level
Author (year)	1	2	3	4	5	6	7	8	Total	%	
Adelantado‐Renau et al. (2019)									6	75.0	High
Bagley and El‐Sheikh (2013)									6	75.0	High
Bagley and El‐Sheikh (2014)									5	62.5	Low
Bottolfs et al. 2020									7	87.5	High
Cardoso et al. (2024)									7	87.5	High
Chaput et al. (2014)									7	87.5	High
Chaput et al. (2023)									7	87.5	High
da Silveira et al. (2018)									6	75.0	High
Dema et al. (2019)									5	62.5	Low
Dong et al. (2023)									7	87.5	High
Dos Santos et al. (2024)									7	87.5	High
Ekstedt et al. (2013)									5	62.5	Low
El‐Sheikh et al. (2007)									7	87.5	High
El‐Sheikh et al. (2019)									6	75.0	High
Eskenazi et al. (2019)									7	87.5	High
Ferranti et al. (2016)									7	87.5	High
Ferrari et al. (2017)									7	87.5	High
Ferrari et al. (2019)									7	87.5	High
Fujimura et al. (2019)									6	75.0	High
Gan et al. (2019)									5	62.5	Low
García‐Hermoso et al. (2017)									7	87.5	High
Golley et al. (2013)									7	87.5	High
Gupta et al. (2002)									5	62.5	Low
Harrex et al. (2018)									5	62.5	Low
Harrington et al. (2021)									6	75.0	High
He et al. (2015)									6	75.0	High
He et al. (2020)									6	75.0	High
Herttrich et al. (2020)									6	75.0	High
Hjorth, Chaput, Damsgaard et al. (2014)									6	75.0	High
Hjorth, Chaput, Gao et al. (2014)									6	75.0	High
Hjorth et al. (2016)									7	87.5	High
Hrafnkelsdottir et al. (2020)									6	75.0	High
Iglayreger et al. (2014)									7	87.5	High
Jankovic et al. (2023)									8	100.0	High
John et al. (2023)									3	32.5	Low
Kathrotia et al. (2010)									6	75.0	High
Kracht et al. (2019)									7	87.5	High
LaVoy et al. (2020)									6	75.0	High
Lemola et al. (2011)									7	87.5	High
Lima et al. (2020)									6	75.0	High
Lucas‐de la Cruz et al. (2018)									7	87.5	High
Lytle et al. (2011)									7	87.5	High
Magalhaes et al. (2020)									5	62.5	Low
Moitra et al. (2020)									6	75.0	High
Moitra et al. (2021)									7	87.5	High
Moore (2007)									6	75.0	High
Morrissey et al. (2019)									6	75.0	High
Negele et al. (2020)									7	87.5	High
Ogutlu et al. (2023)									5	62.5	Low
Olds et al. (2011)									7	87.5	High
Ozkan et al. (2020)									5	62.5	Low
Pabst et al. (2009)									7	87.5	High
Parker et al. (2022)									8	100.0	High
Pickett et al. (2022)									8	100.0	High
Pompeia et al. (2023)									6	75.0	High
Quante, Khandpur et al. 2019									7	87.5	High
Roberto et al. (2023)									7	87.5	High
Rognvaldsdottir et al. (2020)									7	87.5	High
Rosi et al. (2017)									3	37.5	Low
Rosli et al. (2021)									5	62.5	Low
Saleh‐Ghadimi et al. (2019)									5	62.5	Low
Shakir et al. (2018)									7	87.5	High
Stoner et al. (2018)									7	87.5	High
Tabatabaee et al. (2018)									5	62.5	Low
Tee et al. (2018)									7	87.5	High
Wang, Adab et al. (2017)									7	87.5	High
Werneck et al. (2018)									7	87.5	High
Winpenny et al. (2023)									7	87.5	High
Yaghtin et al. (2022)									7	87.5	High

*Note*: Scores a maximum of 8. Green ‐ Yes; Yellow ‐ Unclear; Red ‐ No; Black ‐ Not Applicable.

Abbreviation: JBI, Joanna Briggs Institute.

**TABLE 5:** Quality appraisal of all included longitudinal, non‐randomised controlled interventions and cohort studies using the JBI cohort studies tool.

JBI study quality—Cohort, longitudinal and intervention studies														Quality level
Author (year)	1	2	3	4	5	6	7	8	9	10	11	Total	%	
Bagley et al. (2015)												10	90.9	High
Bates et al. (2016)												6	54.6	Low
Catalan‐Lamban et al. (2023)												9	81.8	High
Goodman et al. (2020)												9	81.8	High
LeMay‐Russell et al. (2021)												7	63.6	Low
Lin et al. (2020)												10	90.9	High
Panagiotou et al. (2023)												7	63.6	Low
Skjakodegard et al. (2021)												7	63.6	Low
Snell et al. (2007)												10	90.9	High
Wang et al. (2024)												7	63.6	Low
Zhang et al. (2024)												10	90.9	High

*Note*: Scores a maximum of 11. Green ‐ Yes; Yellow ‐ Unclear; Red ‐ No; Black ‐ Not Applicable.

Abbreviation: JBI, Joanna Briggs Institute.

### 3.3 Data Synthesis

#### 3.3.1 Sleep Variables

Studies included objective or subjective sleep outcomes, or a combination of both (Table 3). Forty‐nine of the 80 included studies used solely subjective sleep outcomes, 27 studies used objective sleep outcomes (including 26 studies using actigraphy and 1 using polysomnography) and 4 studies used a mixed subjective and objective methodology (Table 3). Sleep variables were grouped into three periods across the night (Gale et al. 2024).

- Pre‐sleep—Sleep health, sleep hygiene, sleep habits, bedtime, sleep onset, latency, sleep onset anxiety, and insomnia symptoms.
- During sleep—Sleep maintenance, fragmentation, efficiency, quality and time in bed.
- Post‐sleep—Waketime, daytime sleepiness and social jetlag.

#### 3.3.2 Obesity and Adiposity Variables

Identified studies included an objective measure of obesity or adiposity (Table 3). Obesity measures included body mass index (BMI) z‐scores (BMIz) and percentiles (BMIp), weight status and body weight. Adiposity measures included body fat percentage, body fat mass, trunk body fat mass, fat mass index, fat‐free mass percentage, waist circumference, hip circumference, a sum of skinfolds, waist‐to‐height ratio and waist‐to‐hip ratio.

#### 3.3.3 Determinant Variables

**FIGURE 2:**
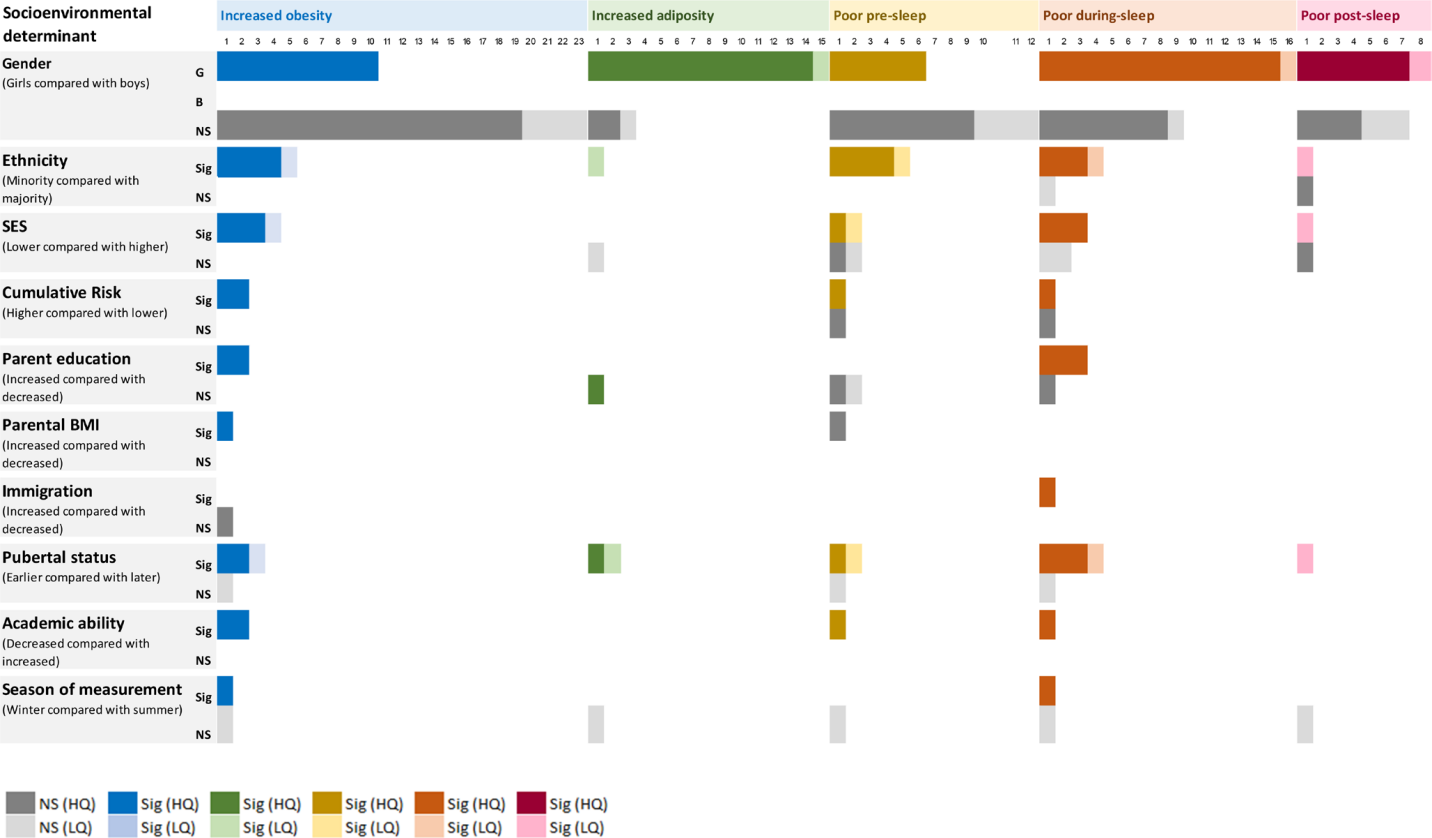
The number of studies showing significant and non‐significant associations between socio‐environmental determinants and obesity, adiposity, pre‐sleep, during‐sleep and post‐sleep variables. Variables were clustered to describe increased obesity, higher adiposity, poor pre‐sleep, poor during‐sleep, and poor post‐sleep. (i) Increased obesity variables included: Increased BMI, BMIz, BMIp, and weight status (overweight or obese). (ii) Higher adiposity variables included a higher body fat percentage only. (iii) Poor pre‐sleep variables included: Later sleep onset and timing, decreased sleep health and increased insomnia symptoms, and bedtime variability. (iv) Poor during‐sleep variables included: Increased time in bed and sleep disturbance, and decreased sleep quality and efficiency. (v) Poor post‐sleep variables included: Later wake time and sleep mid‐point, and increased social jetlag. B – boy; G – girl; HQ – higher‐quality; LQ – lower‐quality; NS – not significant; S – significant. Colour key: Dark grey—non‐significant association (high quality assessment); light grey—non‐significant (low quality assessment); dark blue—significant association between the determinant and increased obesity (high quality assessment); light blue—significant association between the determinant and increased obesity (low quality assessment); dark green—significant association between the determinant and increased obesity (high quality assessment); light green—significant association between the determinant and increased obesity (low quality assessment); dark yellow—significant association between the determinant and increased obesity (high quality assessment); light yellow—significant association between the determinant and increased obesity (low quality assessment); dark orange—significant association between the determinant and increased obesity (high quality assessment); light orange—significant association between the determinant and increased obesity (low quality assessment); dark red—significant association between the determinant and increased obesity (high quality assessment); light red—significant association between the determinant and increased obesity (low quality assessment). Number indicates the number of studies.

**FIGURE 3:**
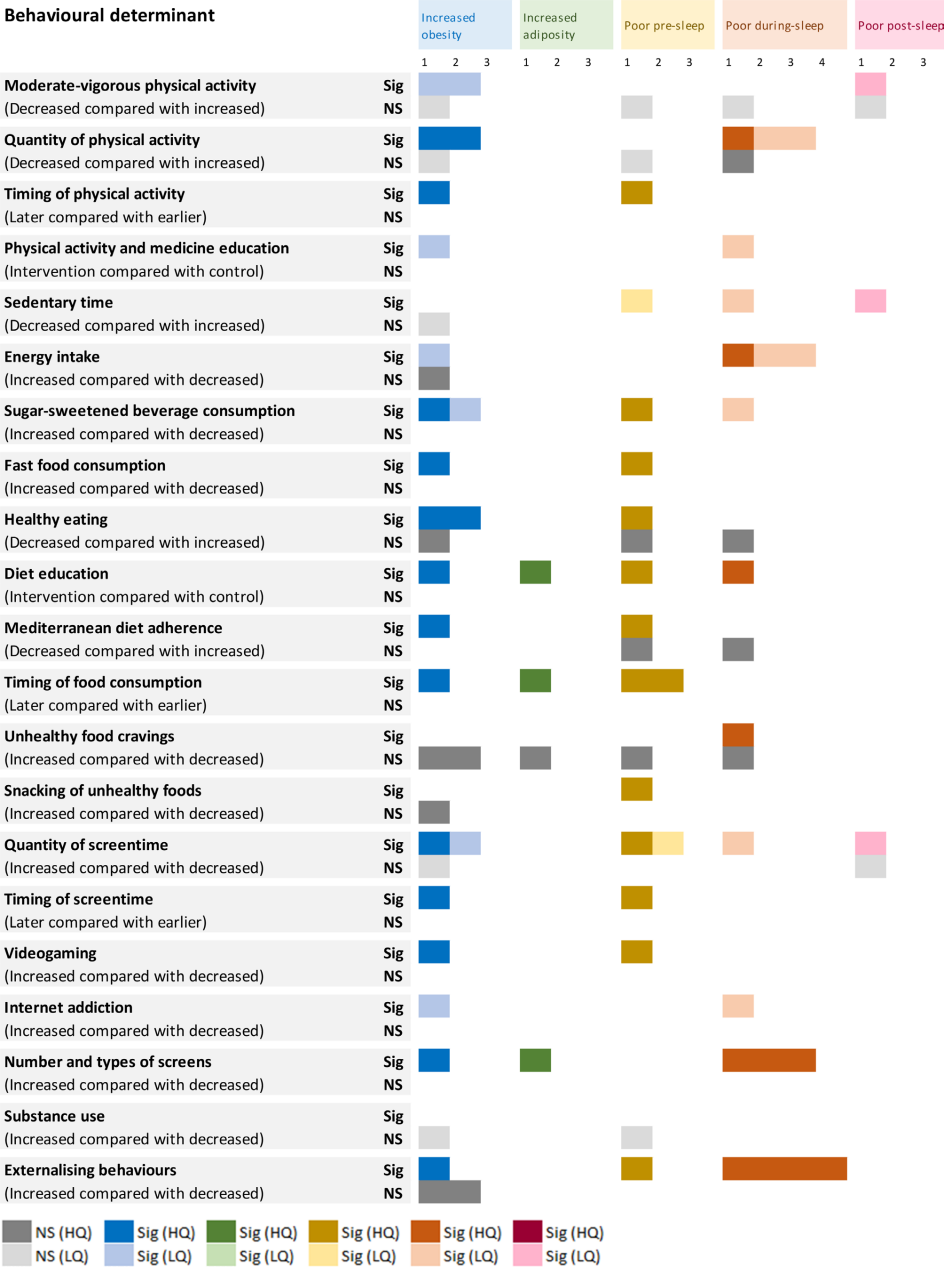
The number of studies showing significant and non‐significant associations between behavioural determinants and obesity, adiposity, pre‐sleep, during‐sleep and post‐sleep variables. Variables were clustered to describe increased obesity, higher adiposity, poor pre‐sleep, poor during‐sleep and poor post‐sleep. (i) Increased obesity variables included: Increased BMI, BMIz, BMIp, and weight status (overweight or obese). (ii) Higher adiposity variables included a higher body fat percentage only. (iii) Poor pre‐sleep variables included: Later sleep onset and timing, decreased sleep health and increased insomnia symptoms, and bedtime variability. (iv) Poor during‐sleep variables included: Increased time in bed and sleep disturbance, and decreased sleep quality and efficiency. (v) Poor post‐sleep variables included: Later wake time and sleep mid‐point, and increased social jetlag. B – boy; G – girl; HQ – higher‐quality; LQ – lower‐quality; NS – not significant; S – significant. Colour key: Dark grey—non‐significant association (high quality assessment); light grey—non‐significant (low quality assessment); dark blue—significant association between the determinant and increased obesity (high quality assessment); light blue—significant association between the determinant and increased obesity (low quality assessment); dark green—significant association between the determinant and increased obesity (high quality assessment); light green—significant association between the determinant and increased obesity (low quality assessment); dark yellow—significant association between the determinant and increased obesity (high quality assessment); light yellow—significant association between the determinant and increased obesity (low quality assessment); dark orange—significant association between the determinant and increased obesity (high quality assessment); light orange—significant association between the determinant and increased obesity (low quality assessment); dark red—significant association between the determinant and increased obesity (high quality assessment); light red—significant association between the determinant and increased obesity (low quality assessment). Number indicates the number of studies.

**FIGURE 4:**
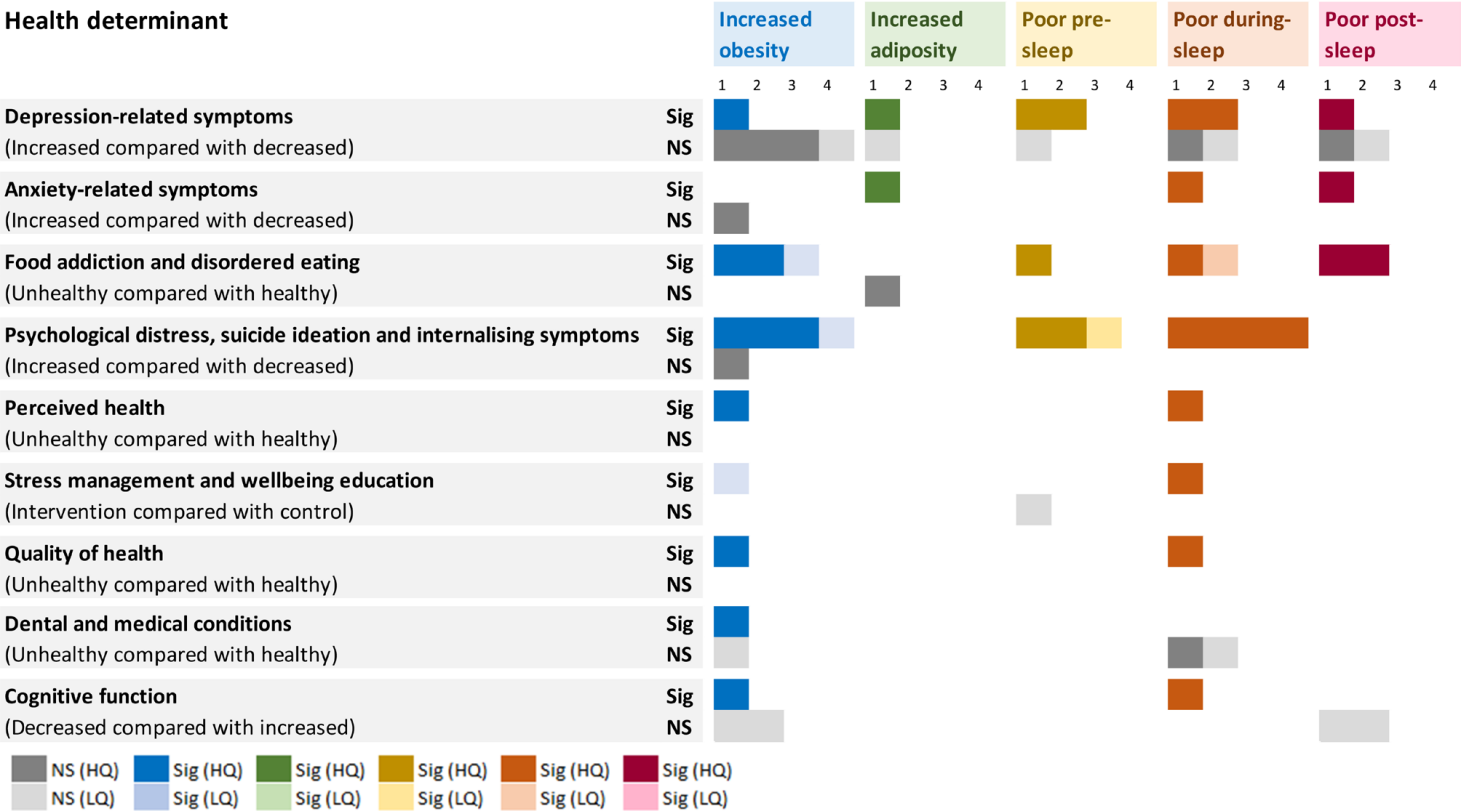
The number of studies showing significant and non‐significant associations between behavioural determinants and obesity, adiposity, pre‐sleep, during‐sleep, and post‐sleep variables. Variables were clustered to describe increased obesity, higher adiposity, poor pre‐sleep, poor during‐sleep, and poor post‐sleep. (i) Increased obesity variables included: Increased BMI, BMIz, BMIp, and weight status (overweight or obese). (ii) Higher adiposity variables included a higher body fat percentage only. (iii) Poor pre‐sleep variables included: Later sleep onset and timing, decreased sleep health and increased insomnia symptoms, and bedtime variability. (iv) Poor during‐sleep variables included: Increased time in bed and sleep disturbance, and decreased sleep quality and efficiency. (v) Poor post‐sleep variables included: Later wake time and sleep mid‐point, and increased social jetlag. Key: HQ – higher‐quality; LQ – lower‐quality; NS – not significant; S – significant. Colour key: Dark grey—non‐significant association (high quality assessment); light grey—non‐significant (low quality assessment); dark blue—significant association between the determinant and increased obesity (high quality assessment); light blue—significant association between the determinant and increased obesity (low quality assessment); dark green—significant association between the determinant and increased obesity (high quality assessment); light green—significant association between the determinant and increased obesity (low quality assessment); dark yellow—significant association between the determinant and increased obesity (high quality assessment); light yellow—significant association between the determinant and increased obesity (low quality assessment); dark orange—significant association between the determinant and increased obesity (high quality assessment); light orange—significant association between the determinant and increased obesity (low quality assessment); dark red—significant association between the determinant and increased obesity (high quality assessment); light red—significant association between the determinant and increased obesity (low quality assessment). Number 1–3 indicates the number of studies.

Studies were then clustered during data synthesis into three different determinant categories: socioenvironmental (47 included studies) (Figure 2), behavioural (26 included studies) (Figure 3) and health determinants (21 included studies) (Figure 4).

#### 3.3.4 Other Analyses

A meta‐analysis was not considered due to the anticipated heterogeneity in sleep outcomes and determinant measures and the age range of the population.

### 3.4 Quality Assessment of the Studies

#### 3.4.1 Cross‐Sectional Studies

Using the JBI cross‐sectional study tool (Moola et al. 2020), the mean quality score and SD were 6.3 ± 1.0 (range 3–8, maximum score 8) (Table 4). Many studies received a nonapplicable score for domain four related to whether control groups were used. The most common threats to quality in cross‐sectional studies were domains 1 (inclusion criteria defined), 5 (confounding variables identified) and 6 (strategies to deal with confounding variables stated). Fifty‐five studies were classified as higher quality, and 14 were considered lower quality.

#### 3.4.2 Cohort, Longitudinal and Non‐Randomised Controlled Intervention Studies

Using the JBI cohort study tool (Moola et al. 2020), the mean quality score and SD were 8.4 ± 1.5 (range 6–10, maximum score 11) (Table 5). A common threat to quality in the studies assessed using the cohort study tool was that multiple studies failed to report domain 6 (whether the participants were free of the outcome at the start of the study) as the studies were recruited using a general population sample. Six studies were classified as higher quality, and five were considered lower quality.

### 3.5 Socioenvironmental Determinants of Sleep, Obesity and Adiposity

Of the 47 included studies that investigated the shared socioenvironmental determinants of sleep, obesity and adiposity, 89.4% were higher‐quality studies, and 10.6% were lower‐quality studies. Figure 2 shows a breakdown of the significant and non‐significant associations between socioenvironmental determinants and sleep, obesity and adiposity by quality level.

#### 3.5.1 Gender

Thirty‐seven studies in this review examined the relationship between gender, sleep, obesity and adiposity (Table 3) (LaVoy et al. 2020; Adelantado‐Renau et al. 2019; Bottolfs et al. 2020; Eskenazi et al. 2019; Ferrari et al. 2017; Ferrari et al. 2019; Fujimura et al. 2019; García‐Hermoso et al. 2017; Golley et al. 2013; Harrex et al. 2018; Hjorth, Chaput, Damsgaard et al. 2014; Hjorth, Chaput, Gao et al. 2014; Hrafnkelsdottir et al. 2020; Iglayreger et al. 2014; Kathrotia et al. 2010; LeMay‐Russell et al. 2021; Lemola et al. 2011; Lima et al. 2020; Lin et al. 2020; Lucas‐de la Cruz et al. 2018; Lytle et al. 2011; Magalhaes et al. 2020; Moitra et al. 2021; Negele et al. 2020; Olds et al. 2011; Ozkan et al. 2020; Rognvaldsdottir et al. 2020; Shakir et al. 2018; Stoner et al. 2018; Wang, Adab et al. 2017; Werneck et al. 2018; Chaput et al. 2023; Pompeia et al. 2023; Roberto et al. 2023; Wang et al. 2024; Winpenny et al. 2023; Zhang et al. 2024).

Twelve studies reported gender‐related differences in obesity variables. Girls had significantly higher BMI (Bottolfs et al. 2020; Fujimura et al. 2019; Negele et al. 2020; Wang, Adab et al. 2017), higher BMIp (Eskenazi et al. 2019), higher BMIz (Iglayreger et al. 2014; Wang, Adab et al. 2017; Chaput et al. 2023), higher body weight (Rognvaldsdottir et al. 2020) and higher weight status (Ferrari et al. 2019; Wang, Adab et al. 2017; Roberto et al. 2023) than boys (Table 3). In contrast, 26 studies reported no significant gender differences in BMI (Adelantado‐Renau et al. 2019; Ferrari et al. 2019; García‐Hermoso et al. 2017; Hrafnkelsdottir et al. 2020; Iglayreger et al. 2014; Kathrotia et al. 2010; Lemola et al. 2011; Lucas‐de la Cruz et al. 2018; Rognvaldsdottir et al. 2020; Stoner et al. 2018; Werneck et al. 2018; Pompeia et al. 2023), BMIp (Lytle et al. 2011), BMIz (LaVoy et al. 2020; Eskenazi et al. 2019; LeMay‐Russell et al. 2021; Lin et al. 2020; Magalhaes et al. 2020; Winpenny et al. 2023), body weight (García‐Hermoso et al. 2017; Lin et al. 2020; Lucas‐de la Cruz et al. 2018; Magalhaes et al. 2020; Stoner et al. 2018) and weight status (Fujimura et al. 2019; García‐Hermoso et al. 2017; Golley et al. 2013; Harrex et al. 2018; Hjorth, Chaput, Damsgaard et al. 2014; Hjorth, Chaput, Gao et al. 2014; Lytle et al. 2011; Moitra et al. 2021; Olds et al. 2011; Ozkan et al. 2020; Stoner et al. 2018) (Table 3).

Fourteen studies reported gender differences in adiposity; girls had a higher body fat percentage (Ferrari et al. 2017; Ferrari et al. 2019; Hjorth, Chaput, Gao et al. 2014; Hrafnkelsdottir et al. 2020; Lucas‐de la Cruz et al. 2018; Lytle et al. 2011; Rognvaldsdottir et al. 2020; Shakir et al. 2018; Stoner et al. 2018; Wang, Adab et al. 2017; Werneck et al. 2018; Pompeia et al. 2023), higher waist circumference (Ferrari et al. 2017; Rognvaldsdottir et al. 2020; Wang, Adab et al. 2017), higher fat mass index (Hjorth, Chaput, Damsgaard et al. 2014; Hjorth, Chaput, Gao et al. 2014; Stoner et al. 2018), higher fat‐free mass index (Hjorth, Chaput, Gao et al. 2014), higher fat mass (Stoner et al. 2018), a higher sum of skinfolds (Lima et al. 2020), higher trunk fat percentage (Rognvaldsdottir et al. 2020), higher waist‐to‐hip ratio (Stoner et al. 2018) and higher waist‐to‐height ratio (Wang, Adab et al. 2017) than boys (Table 3). Ten studies reported no significant gender differences in body fat percentage (Magalhaes et al. 2020), waist circumference (Ferrari et al. 2019; García‐Hermoso et al. 2017; Hjorth, Chaput, Damsgaard et al. 2014; Hjorth, Chaput, Gao et al. 2014; Iglayreger et al. 2014; Lin et al. 2020; Lucas‐de la Cruz et al. 2018; Magalhaes et al. 2020) and waist‐to‐height ratio (Shakir et al. 2018) (Table 3).

Eighteen studies reported on gender differences across sleep variables within the pre‐sleep domain (LaVoy et al. 2020; García‐Hermoso et al. 2017; Harrex et al. 2018; Hrafnkelsdottir et al. 2020; Kathrotia et al. 2010; LeMay‐Russell et al. 2021; Lemola et al. 2011; Lima et al. 2020; Lin et al. 2020; Lucas‐de la Cruz et al. 2018; Lytle et al. 2011; Negele et al. 2020; Olds et al. 2011; Rognvaldsdottir et al. 2020; Shakir et al. 2018; Stoner et al. 2018; Zhang et al. 2024; Wang, Gao et al. 2017). Girls had a significantly higher sleep onset latency (Negele et al. 2020; Zhang et al. 2024), increased severity of insomnia symptom (Lima et al. 2020; Lin et al. 2020), increased sleep onset anxiety (García‐Hermoso et al. 2017), later bedtime (Hrafnkelsdottir et al. 2020), increased bedtime variability (Hrafnkelsdottir et al. 2020; Lytle et al. 2011), later sleep onset (Kathrotia et al. 2010) and poorer sleep habits (LaVoy et al. 2020; García‐Hermoso et al. 2017) than boys (Table 3). Other studies reported no significant gender differences in sleep onset latency (LaVoy et al. 2020; Hrafnkelsdottir et al. 2020; Kathrotia et al. 2010; Lemola et al. 2011; Lucas‐de la Cruz et al. 2018), sleep onset latency variability (Hrafnkelsdottir et al. 2020), bedtime (LeMay‐Russell et al. 2021; Lucas‐de la Cruz et al. 2018; Olds et al. 2011; Rognvaldsdottir et al. 2020; Shakir et al. 2018; Stoner et al. 2018; Wang, Adab et al. 2017; Roberto et al. 2023), bedtime variability (Kathrotia et al. 2010; LeMay‐Russell et al. 2021; Rognvaldsdottir et al. 2020), sleep variability (LeMay‐Russell et al. 2021; Rognvaldsdottir et al. 2020), sleep onset (Harrex et al. 2018; Pompeia et al. 2023) and sleep habits (Hjorth, Chaput, Damsgaard et al. 2014; Hjorth, Chaput, Gao et al. 2014) (Table 3).

Twenty‐four studies reported on gender differences across sleep variables within the during‐sleep domain (LaVoy et al. 2020; Adelantado‐Renau et al. 2019; Bottolfs et al. 2020; Ferrari et al. 2017; Ferrari et al. 2019; Fujimura et al. 2019; García‐Hermoso et al. 2017; Golley et al. 2013; Harrex et al. 2018; Hrafnkelsdottir et al. 2020; Kathrotia et al. 2010; Lemola et al. 2011; Lima et al. 2020; Lucas‐de la Cruz et al. 2018; Lytle et al. 2011; Magalhaes et al. 2020; Negele et al. 2020; Ozkan et al. 2020; Rognvaldsdottir et al. 2020; Wang, Adab et al. 2017; Werneck et al. 2018; Winpenny et al. 2023; Zhang et al. 2024; Ekstedt et al. 2013). Girls had significantly lower sleep quality (Adelantado‐Renau et al. 2019; Fujimura et al. 2019; Lima et al. 2020; Moitra et al. 2021; Ekstedt et al. 2013), lower sleep efficiency (Lemola et al. 2011; Lucas‐de la Cruz et al. 2018; Negele et al. 2020; Zhang et al. 2024), higher sleep efficiency variability (Hrafnkelsdottir et al. 2020), increased sleep disturbance (Bottolfs et al. 2020), increased sleep fragmentation (LaVoy et al. 2020), increased wake after sleep onset (Negele et al. 2020; Zhang et al. 2024), increased awakening variability (Hrafnkelsdottir et al. 2020), longer time in bed (Lucas‐de la Cruz et al. 2018), increased time in bed variability (Hrafnkelsdottir et al. 2020) and later sleep mid‐point (Winpenny et al. 2023; Zhang et al. 2024) than boys (Table 3). In contrast, other studies found no significant gender differences in sleep quality (Ferrari et al. 2017; Ferrari et al. 2019; García‐Hermoso et al. 2017; Hrafnkelsdottir et al. 2020; Kathrotia et al. 2010; Magalhaes et al. 2020; Ozkan et al. 2020; Werneck et al. 2018; Chaput et al. 2023), sleep efficiency (LaVoy et al. 2020; Hrafnkelsdottir et al. 2020; Chaput et al. 2023), sleep disturbance (Wang, Adab et al. 2017), wake after sleep onset (LaVoy et al. 2020; Rognvaldsdottir et al. 2020), sleep timing (Golley et al. 2013; Harrex et al. 2018; Hrafnkelsdottir et al. 2020), sleep variability (LeMay‐Russell et al. 2021; Rognvaldsdottir et al. 2020), sleep regularity (Chaput et al. 2023), time in bed (Hrafnkelsdottir et al. 2020; Rognvaldsdottir et al. 2020), sleep mid‐point (LeMay‐Russell et al. 2021) and night eating (Lytle et al. 2011) (Table 3).

Eleven studies reported on gender differences across sleep variables within the post‐sleep domain (Bottolfs et al. 2020; Harrex et al. 2018; Hrafnkelsdottir et al. 2020; Kathrotia et al. 2010; LeMay‐Russell et al. 2021; Lucas‐de la Cruz et al. 2018; Lytle et al. 2011; Rognvaldsdottir et al. 2020; Shakir et al. 2018; Stoner et al. 2018; Roberto et al. 2023). Girls had a significantly later waketime (Harrex et al. 2018; Hrafnkelsdottir et al. 2020; Kathrotia et al. 2010) (weekend only (Stoner et al. 2018; Roberto et al. 2023)), increased waketime variability (Hrafnkelsdottir et al. 2020), increased social jetlag (Lytle et al. 2011; Stoner et al. 2018; Pompeia et al. 2023) and increased daytime sleepiness (Bottolfs et al. 2020) than boys (Table 3). Other studies reported no significant gender differences in waketime (LeMay‐Russell et al. 2021; Lucas‐de la Cruz et al. 2018; Rognvaldsdottir et al. 2020; Shakir et al. 2018; Pompeia et al. 2023) and waketime variability (Kathrotia et al. 2010; LeMay‐Russell et al. 2021; Lytle et al. 2011) and daytime sleepiness (Kathrotia et al. 2010) (Table 3).

#### 3.5.2 Ethnicity

The relationship between ethnicity, sleep, obesity and adiposity was examined in six studies (He et al. 2015; LeMay‐Russell et al. 2021; Zhang et al. 2024; Gupta et al. 2002; He et al. 2020; Snell et al. 2007). Compared with non‐Hispanic white participants, African American and Hispanic participants had a significantly higher weight status (Gupta et al. 2002) and African American, Hispanic, Asian and American Indian participants had a significantly higher BMIp (He et al. 2015; He et al. 2020) (Table 3). Compared with Caucasian participants, African American and non‐Hispanic white participants had higher fat mass (LeMay‐Russell et al. 2021) and higher BMI (Snell et al. 2007) and those with a minority identification had a significantly higher BMI longitudinally (Zhang et al. 2024; Snell et al. 2007) (Table 3).

Compared with non‐Hispanic white participants, African American, Hispanic, Asian, and American Indian participants had significantly higher sleep variability (He et al. 2015; He et al. 2020) (Table 3). Compared with Caucasian participants, African American and non‐Hispanic white participants had significantly later bedtimes (Snell et al. 2007), waketimes (LeMay‐Russell et al. 2021) and sleep mid‐points (LeMay‐Russell et al. 2021) and those with a minority identification had a significantly lower sleep efficiency (Zhang et al. 2024), increased sleep onset latency (Zhang et al. 2024), increased wake after sleep onset (Zhang et al. 2024) and a later sleep mid‐point (Zhang et al. 2024) (Table 3). In contrast, some studies reported no significant differences between ethnicities in waketimes (Snell et al. 2007), sleep disturbance (Gupta et al. 2002), social jetlag (LeMay‐Russell et al. 2021), bedtime variability (LeMay‐Russell et al. 2021) and waketime variability (LeMay‐Russell et al. 2021) (Table 3).

#### 3.5.3 Socioeconomic Status

Four studies examined the associations between socioeconomic status (SES) and sleep, obesity and adiposity (LeMay‐Russell et al. 2021; Zhang et al. 2024; Snell et al. 2007; Dong et al. 2023) (Table 3). A lower SES was significantly associated with an increased BMI, a later bedtime and waketime, but not with BMI longitudinally (Snell et al. 2007). A significant association was found between a lower SES and a higher BMIz (LeMay‐Russell et al. 2021; Zhang et al. 2024), increased bedtime variability (LeMay‐Russell et al. 2021), increased wake after sleep onset (Zhang et al. 2024), a later sleep mid‐point (Zhang et al. 2024) and increased social jetlag (LeMay‐Russell et al. 2021) but not fat mass (LeMay‐Russell et al. 2021), sleep onset latency (Zhang et al. 2024), sleep efficiency (Zhang et al. 2024), sleep variability (LeMay‐Russell et al. 2021), waketime variability (LeMay‐Russell et al. 2021), bedtime (LeMay‐Russell et al. 2021) or waketime (LeMay‐Russell et al. 2021) (Table 3). Increased food insecurity as a marker for poor SES was found to be significantly associated with BMIz, increased sleep disturbance and a predictor of a higher waist circumference (mediated by poor sleep health) (Dong et al. 2023) (Table 3).

#### 3.5.4 Caregivers Education

Four studies examined the association between caregivers’ education level and sleep, obesity and adiposity (Lima et al. 2020; Lin et al. 2020; Zhang et al. 2024; Snell et al. 2007). A lower parental education level was found to be associated with an increased BMI, lower sleep efficiency, increased wake after sleep onset and a later mid‐point, but not sleep onset latency (Zhang et al. 2024) (Table 3). Significant longitudinal associations were found between lower paternal education and increased child BMIz and increased child insomnia symptoms (Lin et al. 2020) and between a lower caregiver education and increased child BMI (Snell et al. 2007) (Table 3). In contrast, no significant associations were found between maternal education and child sleep quality (Lima et al. 2020) and longitudinally between caregiver education and child BMI, bedtime or waketime (Snell et al. 2007) (Table 3).

#### 3.5.5 Pubertal Status

Four studies measured the association between pubertal status, sleep, obesity and adiposity (LeMay‐Russell et al. 2021; Zhang et al. 2024; Gupta et al. 2002; Herttrich et al. 2020). Significant associations were found between pubertal development and increased weight status (Gupta et al. 2002), increased BMI (Zhang et al. 2024; Herttrich et al. 2020), increased BMIz (Herttrich et al. 2020), increased skinfolds (Herttrich et al. 2020), a larger hip circumference (Herttrich et al. 2020), a larger waist circumference (Herttrich et al. 2020), a larger waist‐to‐hip ratio (Herttrich et al. 2020), increased body fat percentage (Herttrich et al. 2020), increased arousal index score (Herttrich et al. 2020), increased sleep onset latency (Zhang et al. 2024), increased wake after sleep onset (Zhang et al. 2024), later sleep mid‐point (Zhang et al. 2024) but not sleep disturbance (Gupta et al. 2002), sleep efficiency (Zhang et al. 2024) and the apnoea‐hypopnea index (Herttrich et al. 2020) (Table 3). Longitudinally, pubertal status was significantly associated with an increased fat mass, increased sleep variability, increased social jetlag, a later waketime, bedtime and sleep mid‐point (LeMay‐Russell et al. 2021) (Table 3).

### 3.6 Behavioural Determinants of Sleep, Obesity and Adiposity

Out of the 26 included studies that investigated the shared behavioural determinants of sleep, obesity and adiposity, 61.5% were higher‐quality studies, and 38.5% were lower‐quality studies. Figure 3 shows a breakdown of the significant and non‐significant associations between behavioural determinants of sleep, obesity and adiposity by quality level.

#### 3.6.1 Physical Activity and Movement Patterns

##### 3.6.1.1 Moderate‐To‐Vigorous Physical Activity

Three studies measured the association between moderate‐vigorous physical activity (MVPA) and sleep, obesity and adiposity (Ekstedt et al. 2013; Bates et al. 2016; Skjakodegard et al. 2021). Significant associations were found between decreased MVPA and increased weight status (Ekstedt et al. 2013; Skjakodegard et al. 2021), BMI (Ekstedt et al. 2013), sleep mid‐point (Skjakodegard et al. 2021), waketime (Ekstedt et al. 2013) and social jetlag (Skjakodegard et al. 2021), as well as a significant association between decreased MVPA and poorer sleep efficiency (Ekstedt et al. 2013) (Table 3). A significant longitudinal association was found between decreased MVPA and a later sleep onset and between a later waketime and decreased MVPA, but not between MVPA and BMIz, BMIp, sleep onset, and waketime (Bates et al. 2016) (Table 3). A later sleep onset predicted reduced MVPA, and a reduced MVPA predicted poorer sleep efficiency, a later sleep onset, and a later waketime (Ekstedt et al. 2013) (Table 3).

##### 3.6.1.2 Quantity and Timing of Physical Activity

Five studies measured the quantity of physical activity or the timing of physical activity and its association with sleep, obesity and adiposity (Wang et al. 2024; Gupta et al. 2002; Ferranti et al. 2016; Morrissey et al. 2019; Dos Santos et al. 2024). Decreased physical activity was significantly associated with increased sleep disturbance (Gupta et al. 2002), but not sleep timing (Ferranti et al. 2016), sleep quality (Ferranti et al. 2016) or weight status (Gupta et al. 2002) (Table 3). Adolescents who met physical activity guidelines had increased sleep health and decreased weight status (Morrissey et al. 2019) (Table 3). Adolescents with insufficient physical activity and higher levels of screen time, when adjusted for BMI, were significantly associated with poor sleep quality (Dos Santos et al. 2024) (Table 3). An 8‐week physical exercise intervention, combined with integrated sports medicine education, found that both the physical exercise intervention group and the combined intervention group had a significantly higher reduction in BMI and sleep quality compared with the control group (Wang et al. 2024) (Table 3). Increased physical activity taking place in the 1‐h before bed was significantly associated with decreased weight status and increased sleep health (Morrissey et al. 2019) (Table 3).

One study reported that increased sedentary time predicted poorer sleep efficiency, later sleep onset and a later waketime and that later sleep onset and later waketime predicted increased sedentary time (Ekstedt et al. 2013) (Table 3). No significant associations were found between sedentary time and BMI or weight status (Ekstedt et al. 2013) (Table 3).

#### 3.6.2 Diet, Energy Intake and Food Consumption Behaviour

##### 3.6.2.1 Energy Intake

Three studies reported the relationship between energy intake, sleep, obesity and adiposity (Kracht et al. 2019; Saleh‐Ghadimi et al. 2019; Jankovic et al. 2024). A higher energy intake was significantly associated with poorer sleep quality (Saleh‐Ghadimi et al. 2019) and sleep efficiency (Kracht et al. 2019), but not BMI (Saleh‐Ghadimi et al. 2019) (Table 3). A significant association between increased fat intake and poorer sleep quality and between increased carbohydrate intake and increased BMI was found (Saleh‐Ghadimi et al. 2019) (Table 3). No significant associations were identified between BMI and fat or protein intake and between sleep quality and protein or carbohydrate intake (Saleh‐Ghadimi et al. 2019) (Table 3). A later timing of a higher energy intake was found to be significantly associated with a later chronotype, a higher fat mass and a larger change in fat mass (1‐year follow‐up) (Jankovic et al. 2024) (Table 3).

##### 3.6.2.2 Sugar‐Sweetened Beverages (SSBs)

Across two studies (Morrissey et al. 2019; Gan et al. 2019), significant associations were found between increased SSB consumption and poorer sleep health (Morrissey et al. 2019), poorer sleep quality (Gan et al. 2019), increased weight status (Morrissey et al. 2019) and increased BMI (Gan et al. 2019) (Table 3). Sugar‐sweetened beverage consumption (in the 1‐h before bed) was significantly associated with poorer sleep health but not weight status (Morrissey et al. 2019) (Table 3).

##### 3.6.2.3 Fast‐Food Consumption

In one study, a significant association was found between takeaway consumption and poorer sleep health and increased weight status (Morrissey et al. 2019) (Table 3).

##### 3.6.2.4 Healthy Eating and Nutrition

Four studies assessed the associations between healthy eating, sleep, obesity, and adiposity (Morrissey et al. 2019; Kracht et al. 2019; Cardoso et al. 2024; Catalan‐Lamban et al. 2023). One study reported a significant association between healthy eating and a lower weight status and an earlier chronotype (Cardoso et al. 2024), but another study reported no significant association between healthy eating and sleep efficiency or BMI (Kracht et al. 2019) (Table 3). Increased fruit and vegetable consumption was significantly associated with a higher weight status but not sleep health in one study (Morrissey et al. 2019) (Table 3).

A nutrition education intervention reported that 2 years post‐intervention the intervention group had a significantly lower BMI, lower body fat percentage, smaller waist circumference, higher sleep efficiency, decreased sleep onset latency, decreased wake after sleep onset and shorter awakenings across the night, compared with controls (Catalan‐Lamban et al. 2023) (Table 3).

##### 3.6.2.5 Mediterranean Diet

Three studies measured the association between a Mediterranean diet, sleep, obesity and adiposity (Ferranti et al. 2016; Rosi et al. 2017; Yaghtin et al. 2022). Mediterranean diet adherence was significantly associated with decreased insomnia symptoms (Yaghtin et al. 2022), decreased waist circumference (Yaghtin et al. 2022) and BMI (Ferranti et al. 2016), but not with BMIp (Yaghtin et al. 2022), weight status (Rosi et al. 2017), sleep timing (Ferranti et al. 2016) or sleep quality (Ferranti et al. 2016) (Table 3).

##### 3.6.2.6 Food Cravings

Across two studies (Kracht et al. 2019; Parker et al. 2022), increased food cravings (high fats, sweets and starch) were significantly associated with poorer sleep efficiency but not BMI (Parker et al. 2022), fat mass (Kracht et al. 2019) height (Kracht et al. 2019), sleep mid‐point (Kracht et al. 2019), sleep onset (Kracht et al. 2019) or waketime (Kracht et al. 2019) (Table 3).

##### 3.6.2.7 Snacking

Two studies examined the relationship between snacking, sleep, obesity and adiposity (Roberto et al. 2023; Morrissey et al. 2019). A significant association was found between increased snacking and poorer sleep health but not weight status (Morrissey et al. 2019) (Table 3). Another study reported associations between snacking later in the evening and a higher weight status and later chronotype (Roberto et al. 2023) (Table 3).

#### 3.6.3 Screen Time, Videogaming, Internet and Social Media Use

##### 3.6.3.1 Quantity and Timing of Screen Time

Three studies assessed the associations between screentime, sleep, obesity and adiposity (Skjakodegard et al. 2021; Morrissey et al. 2019; John et al. 2023). A significant association was found between increased screen time and a later bedtime (weekday and weekend) (John et al. 2023), later wake time (weekend) (John et al. 2023) and a later sleep mid‐point (Skjakodegard et al. 2021), but not BMI (John et al. 2023), weight status (Skjakodegard et al. 2021) or social jetlag (Skjakodegard et al. 2021) (Table 3). Meeting screen time guidelines was significantly associated with decreased weight status and increased sleep health (Morrissey et al. 2019) (Table 3). A significant association was found between screen time (in the 1‐h before bed) increased weight status and poorer sleep health and between screen time in bed and poorer sleep health (Morrissey et al. 2019) (Table 3).

##### 3.6.3.2 Videogaming

A significant association was found between excessive video game usage and increased BMIz and increased bedtime variability in one study (Goodman et al. 2020) (Table 3).

##### 3.6.3.3 Internet Addiction

Internet “addiction”, as it was referred to in the study by Tabatabaee et al. 2018, was shown to predict increased BMI directly and indirectly (via poor sleep quality, decreased physical activity and increased fast food consumption) in one study (Tabatabaee et al. 2018) (Table 3).

##### 3.6.3.4 Number and Type of Screens

Two studies assessed the associations between screentime, sleep, obesity and adiposity (Chaput et al. 2014; Harrington et al. 2021). A significant association was found between a higher number of accessible screens to the adolescent and increased BMI and decreased time in bed (Harrington et al. 2021) (Table 3). A significant association was shown between a higher number of accessible screens for the adolescent and poorer sleep efficiency, increased weight status and increased body fat percentage and between increased TV usage and increased body fat percentage (Chaput et al. 2014) (Table 3).

#### 3.6.4 Externalising Behaviour

Across three studies (Zhang et al. 2024; El‐Sheikh et al. 2007; El‐Sheikh et al. 2019), significant associations were reported between increased externalising problems and increased BMI (Zhang et al. 2024), poorer sleep efficiency (Zhang et al. 2024; El‐Sheikh et al. 2007), later sleep mid‐point (Zhang et al. 2024) and increased wake after sleep onset (Zhang et al. 2024) and between poorer sleep quality and increased rule‐breaking behaviour and aggressive behaviour (El‐Sheikh et al. 2019) (Table 3). No significant correlations between externalising problems and BMI (El‐Sheikh et al. 2007) and sleep onset latency (Zhang et al. 2024), rule‐breaking behaviour and BMIz (El‐Sheikh et al. 2019) or aggressive behaviour and BMIz were found (El‐Sheikh et al. 2019) (Table 3).

### 3.7 Health Determinants of Sleep, Obesity and Adiposity

Out of the 21 included studies that investigated the shared health determinants of sleep, obesity and adiposity, 66.7% were higher‐quality studies and 33.3% were lower‐quality studies. Figure 4 shows a breakdown of the significant and non‐significant associations between health determinants of sleep, obesity and adiposity by quality level.

#### 3.7.1 Depressive Symptoms

Eight studies assessed the association between depressive symptoms and sleep, obesity and adiposity (LeMay‐Russell et al. 2021; Lima et al. 2020; Lin et al. 2020; Moitra et al. 2021; El‐Sheikh et al. 2007; Moitra et al. 2020; Moore 2007; Pabst et al. 2009). Increased depressive symptoms were shown to predict increased skinfolds (Lima et al. 2020) and decreased sleep quality (Lima et al. 2020) and daytime sleepiness (Moore 2007) were shown to predict increased depressive symptoms, but BMIp was not a predictor of depressive symptoms (Moore 2007) (Table 3). Significant associations were found between increased depressive symptoms and increased weight status (Moitra et al. 2021), increased insomnia symptoms (Lin et al. 2020) and a later chronotype (Pabst et al. 2009) (Table 3). No significant associations were found between depressive symptoms and fat mass (LeMay‐Russell et al. 2021), BMIz (LeMay‐Russell et al. 2021), BMI (El‐Sheikh et al. 2007), weight status (Pabst et al. 2009), sleep efficiency (El‐Sheikh et al. 2007), sleep variability (LeMay‐Russell et al. 2021), bedtime variability (LeMay‐Russell et al. 2021), waketime variability (LeMay‐Russell et al. 2021), social jetlag (LeMay‐Russell et al. 2021), bedtime (LeMay‐Russell et al. 2021), waketime (LeMay‐Russell et al. 2021), sleep mid‐point (LeMay‐Russell et al. 2021) and daytime sleepiness (Moitra et al. 2020) (Table 3).

#### 3.7.2 Anxiety‐Related Symptoms

Two studies assessed associations between anxiety symptoms and sleep, obesity and adiposity (Lima et al. 2020; Moore 2007). Increased anxiety symptoms were shown to predict poorer sleep quality and increased skinfolds (Lima et al. 2020) and daytime sleepiness was shown to predict increased anxiety symptoms (Moore 2007), but BMIp did not predict anxiety symptoms (Moore 2007) (Table 3).

#### 3.7.3 Stress‐Related Symptoms and Quality of Life

One study assessed the effectiveness of an 8‐week self‐awareness, stress and quality of life management lifestyle and education programme (including mindfulness techniques and lifestyle (healthy eating sleep, physical activity and screen time) education) (Panagiotou et al. 2023). Post‐intervention, participants had a significantly lower BMIz compared with baseline, but bedtime did not markedly change (Panagiotou et al. 2023) (Table 3).

#### 3.7.4 Food Addiction and Disordered Eating

Four studies measured associations between food addiction or disordered eating with sleep, obesity and adiposity (Lin et al. 2020; Saleh‐Ghadimi et al. 2019; Parker et al. 2022; Pickett et al. 2022). Significant associations between increased food addiction and a higher BMIz (Lin et al. 2020), increased weight status (Pickett et al. 2022), increased daytime sleepiness (Pickett et al. 2022) and increased insomnia symptoms (Lin et al. 2020) were reported (Table 3). Increased food addiction was shown to predict increased insomnia symptoms and increased BMIz (Lin et al. 2020) (Table 3). Significant associations were reported between eating disorder attitudes, increased insomnia symptoms and increased BMIz (Lin et al. 2020) (Table 3). Increased eating disorder tendencies were shown to predict increased BMIz (Lin et al. 2020) (Table 3). Uncontrollable eating was significantly associated with a later sleep mid‐point and a later waketime, but not sleep onset, fat mass or height (Parker et al. 2022) (Table 3). Decreased sleep quality and increased BMI were shown to predict increased emotional eating (Saleh‐Ghadimi et al. 2019) (Table 3).

#### 3.7.5 Psychological Distress, Suicide Ideation and Internalising Symptoms

Five studies assessed associations between psychological stress, suicide ideation, or internalising problems with sleep, obesity and adiposity (Lin et al. 2020; Zhang et al. 2024; El‐Sheikh et al. 2007; El‐Sheikh et al. 2019; Dema et al. 2019). Significant longitudinal associations were found between increased psychological distress and increased BMIz and increased insomnia symptoms (Lin et al. 2020) (Table 3). Psychological distress was shown to predict increased insomnia symptoms, food addiction and eating disorder behaviours, which predicted increased BMIz (Lin et al. 2020) (Table 3). Significant associations were found between suicide ideation and increased weight status and increased sleep onset anxiety (Dema et al. 2019) (Table 3). Significant associations were found between increased internalising problems and a higher BMI (Zhang et al. 2024), poorer sleep efficiency (Zhang et al. 2024; El‐Sheikh et al. 2007), a later sleep mid‐point (Zhang et al. 2024), increased wake after sleep onset (Zhang et al. 2024) and poorer sleep quality (El‐Sheikh et al. 2019), but not sleep onset latency (Zhang et al. 2024) (Table 3). Internalising problems were shown to predict BMIz and sleep quality (El‐Sheikh et al. 2019) (Table 3).

#### 3.7.6 Cognitive Function

Three studies assessed cognitive function and its association with sleep, obesity and adiposity (Rosli et al. 2021; Tee et al. 2018; Ogutlu et al. 2023). Increased neurocognitive inhibition and poorer working memory were significantly associated with a higher BMIp, increased sleep efficiency and increased sleep quality (Tee et al. 2018) (Table 3). No significant associations were found between cognitive flexibility and BMIz, sleep efficiency or sleep quality (Tee et al. 2018) and between cognitive function and BMIz or daytime sleepiness (Rosli et al. 2021) (Table 3). Sluggish cognitive tempo and cognitive inattention were significantly associated with excessive daytime sleepiness, but not BMI (Ogutlu et al. 2023) (Table 3).

## 4 Discussion

### 4.1 Overall Findings

In this systematic review of 10 databases, 80 studies investigating shared determinants of sleep and obesity or adiposity were identified and synthesised. Of these, 47 studies assessed shared socioenvironmental determinants, 26 assessed shared behavioural determinants and 21 assessed shared health determinants of sleep and obesity or adiposity in 8–18 years. Despite the large number of studies included in this review, most determinants (excluding gender) had previously been assessed in only a small number of studies, indicating gaps in the current literature exploring the shared determinants of sleep and obesity or adiposity in adolescents.

**FIGURE 5:**
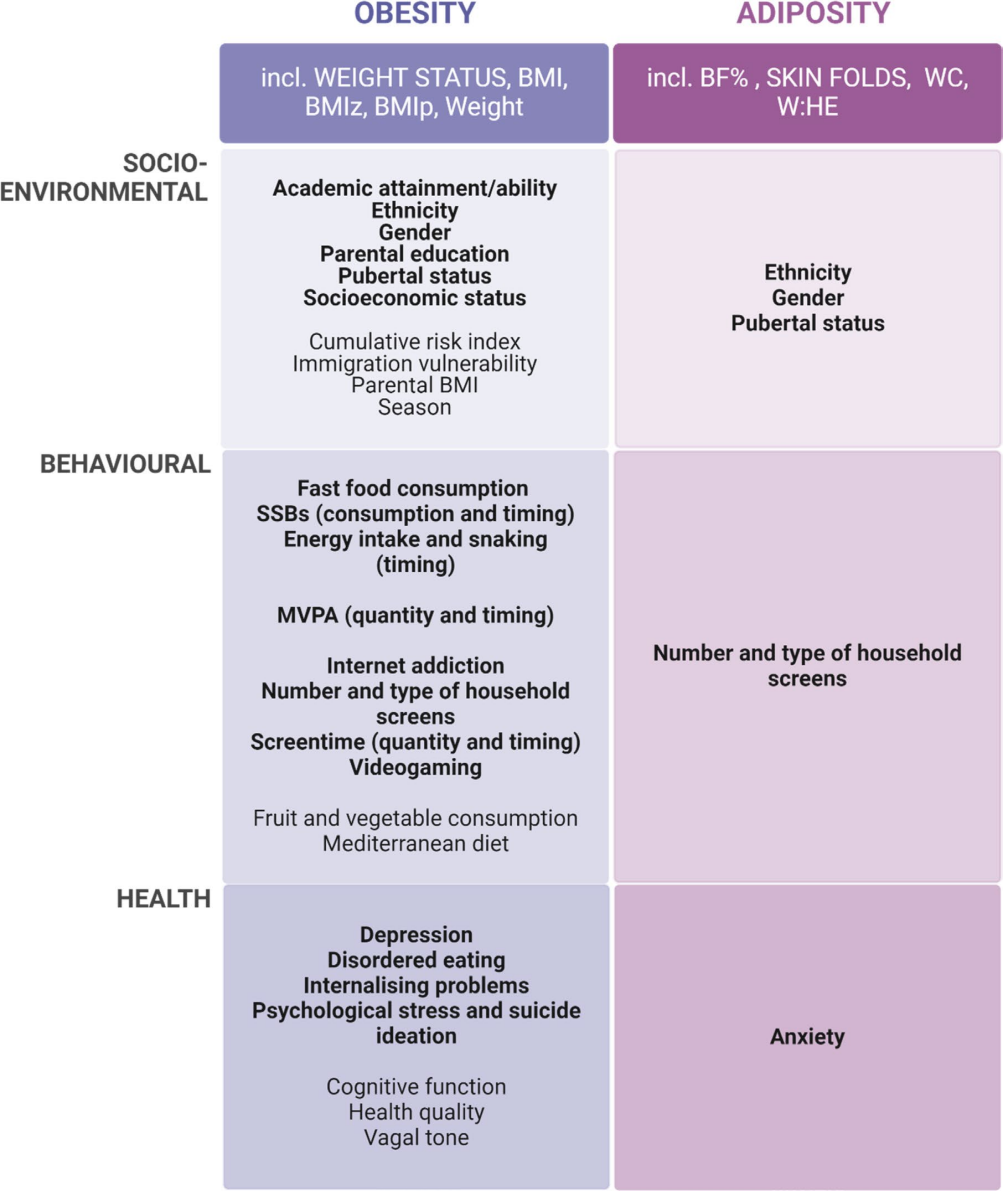
Socioenvironmental, behavioural and health determinants that have shown significant associations with obesity and adiposity in adolescents. Bold font indicates a shared determinant with sleep measures that exhibit consistent significant findings. BF%—body fat percentage; BMI – Body mass index; BMIp – Body mass index percentile; BMIz – Body mass index z‐scores; MVPA – Moderate‐vigorous physical activity; SSB‐sugar‐sweetened beverages; WC – waist circumference; W:He – Waist‐to‐height ratio.

**FIGURE 6:**
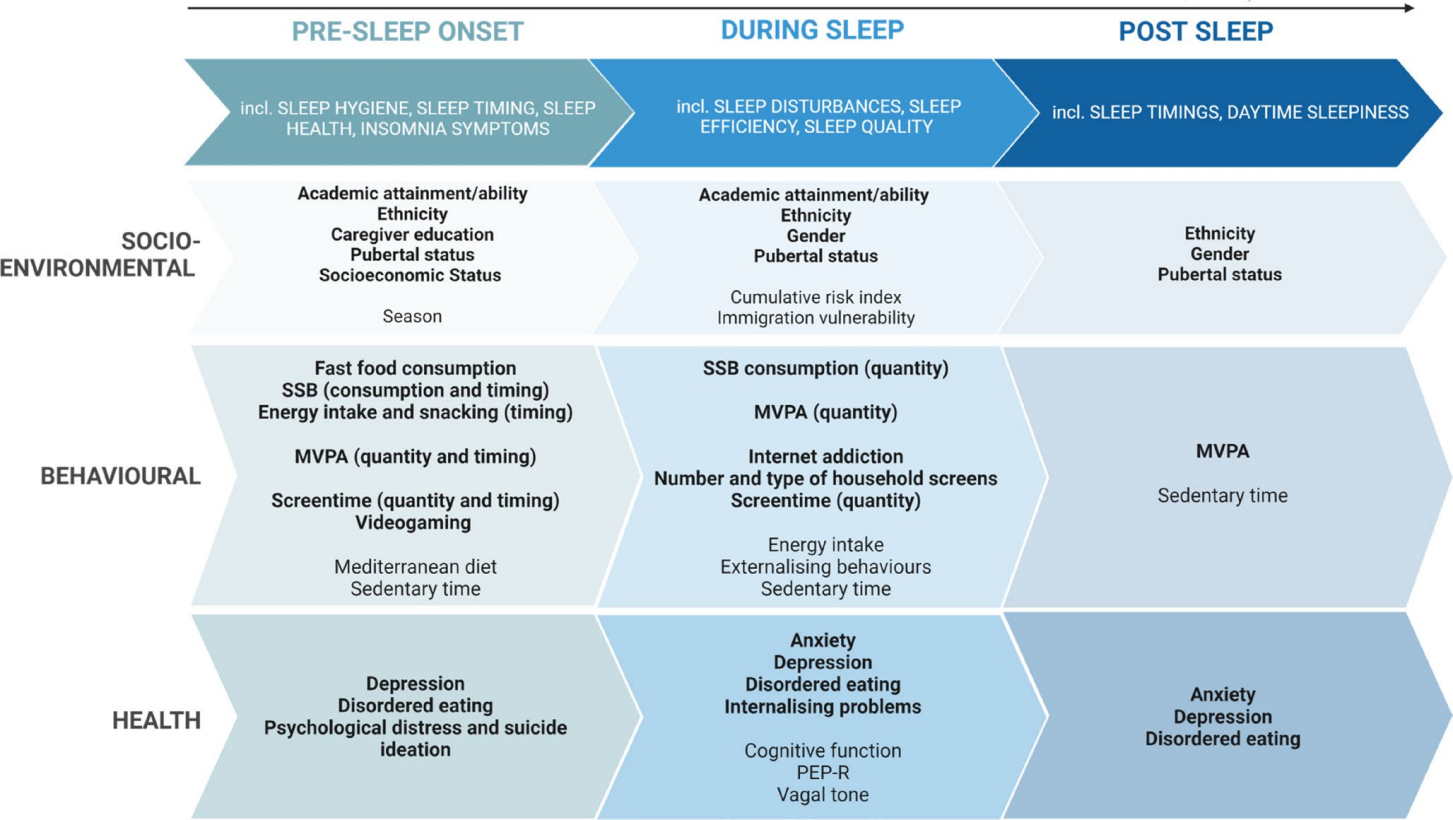
Socioenvironmental, behavioural and health determinants that have shown significant associations with sleep in adolescents. Bold font indicates a shared determinant with obesity and adiposity measures that exhibited consistent significant findings. MVPA – moderate‐vigorous physical activity; PEP‐R – pre‐ejection period regulation; SSB – sugar‐sweetened beverage.

The main findings of this systematic review suggest there are multiple shared socioenvironmental, behavioural and health determinants of adiposity or obesity (Figure 5) and sleep (Figure 6) in adolescents. Behavioural determinants are modifiable and could be targets for health‐promoting interventions to improve adolescent sleep, obesity and adiposity. More studies included in this review measured obesity as an outcome rather than adiposity, highlighting that adiposity measures could be a focus of future research to better understand the shared determinants of sleep and adiposity. There were mixed findings in the socioenvironmental determinant associations with sleep and obesity.

### 4.2 Socioenvironmental Determinants of Poor Sleep, Obesity, and Higher Adiposity

Socioenvironmental determinants were the most thoroughly researched determinants compared with behavioural and health determinants. This could be due to it being less complex to collect socioenvironmental data compared with behavioural and health determinants (Fernandez et al. 2016) and the need to adjust for socioenvironmental determinants in statistical analysis. The most researched socioenvironmental determinants included in the studies in this systematic review were gender (73.2% of the associations between socioenvironmental determinants and sleep and obesity reported), followed by ethnicity (9.4% of the associations between socioenvironmental determinants and sleep and obesity reported) (Figure 2).

A limitation of existing research examining the relationship between sleep and obesity highlighted that there was a large variation in confounders that are adjusted for in analyses (Gale et al. 2024). The shared non‐modifiable socioenvironmental determinants highlighted in the current systematic review should be considered potential confounders and adjusted for in analyses assessing sleep and obesity outcomes. Moreover, these shared socioenvironmental determinants should be considered when designing studies (selecting the sample population, establishing inclusion and exclusion criteria for recruitment) (Hall 2022).

In studies that were scored as higher quality (Figure 2), several socioenvironmental variables were shared determinants of poor sleep and obesity or increased adiposity. Gender was a notable factor, with 55.3% of associations indicating that girls experienced significantly worse sleep, obesity and adiposity outcomes than boys. Ethnicity was also significant, as 91.7% of associations reported worse sleep and obesity outcomes in minority groups compared to majority populations. High cumulative risk scores were associated with poorer outcomes in 66.7% of associations, highlighting the impact of cumulative risk on sleep and obesity. Pubertal status played a universal role, with 100% of associations showing significantly worse sleep, obesity and adiposity outcomes among adolescents experiencing puberty compared to their pre‐pubertal counterparts. Academic ability and attainment also influenced outcomes, with 100% of associations linking lower academic ability to poorer sleep and obesity measures. Finally, the season of measurement was a determinant, with 100% of associations reporting significantly worse sleep and obesity outcomes in individuals measured during the winter months compared to the summer months.

#### 4.2.1 Gender

Gender was found to be more consistently significantly associated with adiposity measures than obesity measures (LaVoy et al. 2020; Adelantado‐Renau et al. 2019; Bottolfs et al. 2020; Eskenazi et al. 2019; Ferrari et al. 2017; Ferrari et al. 2019; Fujimura et al. 2019; García‐Hermoso et al. 2017; Golley et al. 2013; Harrex et al. 2018; Hjorth, Chaput, Damsgaard et al. 2014; Hjorth, Chaput, Gao et al. 2014; Hrafnkelsdottir et al. 2020; Iglayreger et al. 2014; Kathrotia et al. 2010; LeMay‐Russell et al. 2021; Lemola et al. 2011; Lima et al. 2020; Lin et al. 2020; Lucas‐de la Cruz et al. 2018; Lytle et al. 2011; Magalhaes et al. 2020; Moitra et al. 2021; Negele et al. 2020; Olds et al. 2011; Ozkan et al. 2020; Rognvaldsdottir et al. 2020; Shakir et al. 2018; Stoner et al. 2018; Wang, Adab et al. 2017; Werneck et al. 2018). One reason could be that BMIz and BMIp are already adjusted for age and sex, whereas adiposity measures tend not to be (Anderson et al. 2017). In addition, BMI measures are considered “noisy” and are not an accurate measure of body fat distribution (Burkhauser and Cawley 2008). Thus, inclusion of a range of additional adiposity measures would ensure clarity on the determinants of body composition comprehensively.

Gender differences were also found to be consistently significantly associated with three sleep components: the variability of sleep (sleep efficiency, sleep onset latency and time in bed), sleep hygiene (including sleep habits, sleep routine, sleep onset anxiety, insomnia symptoms and social jetlag) and sleep efficiency (efficiency, awakenings and disturbances). These findings support other research indicating that gender differences exist among adolescent sleep hygiene practice (Galland et al. 2017). In younger populations, sleep beliefs, attitudes and knowledge could contribute to gender differences observed during puberty, when a shift to a later chronotype occurs (Díaz‐Morales et al. 2012). When assessing sleep hygiene knowledge in adolescents, gender differences have also been observed (Galland et al. 2017). Female adolescents are reportedly more receptive to educational programmes about sleep hygiene and retain sleep hygiene information longer than male adolescents (Bakotić et al. 2009), although research in this area is in its infancy. Therefore, gender‐specific sleep hygiene education interventions could be considered, and gender should be adjusted for when assessing associations of adiposity, obesity and sleep.

#### 4.2.2 Ethnicity, SES and Caregivers' Education

Similar to gender differences, ethnicity differences were found to be consistently significantly associated with adiposity, pre‐sleep (sleep routine and sleep hygiene) and obesity measures. Thus, like gender, ethnicity should be considered when designing an intervention surrounding sleep routines and sleep hygiene and also when targeting the shared determinants of sleep and obesity in a health‐promoting intervention (Nam et al. 2018). Previous studies have shown significant differences in the attitudes and beliefs of different communities based on ethnicity, SES and geographical location (Nam et al. 2018; Grandner et al. 2013; Rottapel et al. 2020). To the authors’ knowledge, no research considering the differences in ethnicities in sleep routines when designing an intervention has been conducted and thus collecting local sleep behaviour information may be warranted before designing an intervention for that demographic.

Findings from this review showed SES, social jetlag, bedtime variability and obesity were associated. The modification of interventions to account for social and cultural differences in lower‐income families, with ethnicity and gender, should be considered when designing health‐promoting interventions (Rottapel et al. 2020; Quante, Khandpur et al. 2019; Uebergang et al. 2017). Qualitative studies to assess barriers to sleep hygiene practice in specific populations (gender, ethnicity, SES, caregivers education) should be conducted to understand how to tailor a health‐promoting intervention for a subset of the population (Quante, Khandpur et al. 2019). Furthermore, adapting sleep hygiene and obesity interventions to include the family has been shown to be more effective than interventions including the individual alone (Halal and Nunes 2014; Kitzmann and Beech 2011), interventions should be accessible and caregivers' education (including literacy) should considered in their design (Bonuck et al. 2016; Davison et al. 2013). Notably, the number of studies identified in this review measuring SES and caregivers' education was few; thus, more research investigating the associations with during‐sleep outcomes, post‐sleep outcomes, and adiposity is warranted.

#### 4.2.3 Pubertal Status

An earlier pubertal timing was the only socioenvironmental determinant included in this review that was consistently identified as a shared determinant of adiposity, obesity, pre‐sleep, during and post‐sleep. Despite this, only ten studies adjusted for pubertal status when exploring shared determinants and obesity, adiposity and sleep (Adelantado‐Renau et al. 2019; Hjorth, Chaput, Gao et al. 2014; He et al. 2020; Jankovic et al. 2024; Yaghtin et al. 2022; Moore 2007; Pabst et al. 2009; Bagley and El‐Sheikh 2013; Bagley et al. 2015; Quante, Wang et al. 2019). Puberty has been described as a “window of opportunity” for managing the impact on health later in life (Dorn et al. 2019). It has been suggested that an earlier pubertal timing in girls is associated with a higher adult BMI and a later pubertal timing in boys is associated with increased screen time and poor sleep later life (Hoyt et al. 2020). Thus, the pre‐pubertal or early pubertal period may be a good target demographic for a health‐promoting intervention.

#### 4.2.4 Academic Attainment and Ability

This review included a low number of studies assessing academic attainment as a shared determinant of weight status, pre‐sleep outcomes and sleep quality. Previous literature has reported relationships between poor academic attainment, increased screen time, decreased MVPA and obesity (Faught et al. 2017; Marciano and Camerini 2021; García‐Hermoso and Marina 2017; Aguilar et al. 2015). However, little research has been conducted on the interaction between academic ability and attainment and obesity and sleep collectively. Therefore, more research should be conducted in this area to help develop the understanding of how child academic attainment and ability could be adjusted for in population data analyses or what should be considered if recruiting sample populations from specific education institutions (e.g., public, private, state, home‐schooled) (Larsen et al. 2022).

### 4.3 Behavioural Determinants of Poor Sleep, Obesity and Higher Adiposity

The shared behavioural determinants identified in this systematic review can be considered modifiable targets in health‐promoting intervention programmes to improve sleep and reduce adiposity in adolescents.

The quality of the studies included in this review that investigated behavioural shared determinants was lower than that of studies assessing socioenvironmental and health shared determinants; only 60.9% of behavioural determinant associations reported came from high‐quality studies (Figure 3). Furthermore, many of the behavioural determinants identified in this systematic review were explored in only a small number (one to four) of studies (Figure 3). Thus, more robust studies need to be completed to understand and confirm the shared behavioural determinants of sleep and obesity.

When considering only the higher‐quality studies (Figure 3), the findings of this systematic review have indicated that the shared determinants of sleep and obesity or adiposity were: later timing of physical activity, more frequent SSB consumption, more frequent fast‐food consumption, poor adherence to the Mediterranean diet, higher quantity of screen time, late‐night screen time usage, higher videogame usage, and having access to a higher number of screens.

#### 4.3.1 Physical Activity and Movement Patterns

Most studies included in this systematic review that assessed physical activity as a shared determinant of sleep and obesity or adiposity were of low quality. When considering all studies included in this systematic review (regardless of study quality), a lower quantity of MVPA was identified as a shared behavioural determinant of poorer sleep timings (including increased social jetlag) and obesity (Ekstedt et al. 2013; Bates et al. 2016; Skjakodegard et al. 2021). Noticeably, the quantity of MVPA, rather than the quantity of physical activity (including non‐MVPA) or sedentary time, appears to contribute to both sleep and obesity. Adiposity was rarely measured in the included studies, and the MVPA associations were limited to weight status and BMI measures. Exercise interventions (increasing the quantity and regularity of MVPA) have been found to increase sleep efficiency (McDonough et al. 2022) and reduce adiposity (Robbins et al. 2020) and the prevalence of obesity (Hollis et al. 2016). Participating in physical activity, particularly sports, declines throughout adolescence (girls earlier than boys) and consequently, exercise interventions should target the pre‐pubertal or early adolescence period (Farooq et al. 2020).

Only one study (albeit a higher‐quality study) investigating late‐night physical activity was identified as a shared determinant of poor sleep and obesity (Morrissey et al. 2019). Previous research has shown that physical activity in the evening can be beneficial for subsequent health (Janssen et al. 2022). Previous systematic review evidence has indicated that MVPA late in the evening prior to bed is associated with reduced subsequent sleep onset latency and reduced insomnia symptoms (Pesonen et al. 2011; Flausino et al. 2012), implying that timing (a small window of time in the evening) might be important for optimising health. Consequently, an intervention addressing MVPA quantity, regularity and timing during the day could maximise the health benefits gained from an intervention and thus could be a promising area for future research.

#### 4.3.2 Diet, Energy Intake and Food Consumption Behaviour

The findings relating to energy intake and diets as potential shared determinants for sleep, obesity, and adiposity were mixed. This may be due to there being few studies included in this review for each determinant variable and a range of low‐ and high‐quality studies (Figure 3). However, when considering only high‐quality studies, unhealthy diet choices (increased SSBs consumption, later consumption timing and increased fast‐food consumption) were consistently identified as a shared determinant of poorer pre‐sleep health, poorer sleep hygiene and obesity.

The timing and regularity of consumption of SSBs and unhealthy food choices are a novel area of research, and investigating the association with sleep (Morrissey et al. 2019; Goodman et al. 2020), adiposity, and obesity more thoroughly using longitudinal studies would help indicate if the timing and regularity of consumption should be targeted for a health‐promoting intervention. Previous research also suggests the emerging importance of a combined approach of sleep hygiene education, physical activity programmes and healthy dietary choices would be beneficial for individuals who are overweight or living with obesity and are trying to lose weight (Hall 2022). Two interventions investigating the efficacy of sleep hygiene education programmes combined with physical exercise and a healthy diet have led to improved weight status and reduced obesity in adults (Briguglio et al. 2020; Wilson et al. 2022). Similar studies should be conducted in the adolescent population.

#### 4.3.3 Screen Time, Videogaming, Internet and Social Media Use

Screen time (increased quantity and later timing), increased videogaming and a higher number and type of household screens were identified as shared determinants of sleep and adiposity or obesity (Skjakodegard et al. 2021; Morrissey et al. 2019; Goodman et al. 2020; Tabatabaee et al. 2018; Chaput et al. 2014; Harrington et al. 2021).

Like MVPA and unhealthy dietary choices, screen time (increased quantity and later timing) and increased videogaming were associated with increased obesity and poorer pre‐sleep measures such as reduced sleep health and poorer sleep hygiene (Skjakodegard et al. 2021; Morrissey et al. 2019; Goodman et al. 2020). Screen time and videogaming are consistently reported as impacting sleep regularity (Stiglic and Viner 2019) and poorer dietary choices, later and irregular timing of diet and consequently, the development of obesity (Shqair et al. 2019). A health‐promoting intervention programme, including minimising screen time and videogaming, should improve sleep and obesity in adolescents (Jones et al. 2021). The quantity of videogaming has been associated with poor sleep hygiene, additional sleep measures, obesity and adiposity (Goodman et al. 2020); however, the timing and type of videogaming have yet to be examined in association with sleep, obesity and adiposity.

Previous research has reported multiple potential barriers to reducing adolescents' screen time. For example, many schools have adapted online learning platforms for in‐class and outside‐of‐class use, increasing academic work screen time, particularly during and post‐COVID‐19 (Seguin et al. 2021). Additionally, many adolescents have reported feeling connected to friends and that they would feel “lost” or “lonely” without their internet connection to friends (Jones et al. 2021). Implementing screen time restrictions into a health‐promoting intervention would therefore need to consider carefully how other aspects of an individual's health, such as anxiety, stress, loneliness and loss of social connections, might be affected. Several meta‐analyses have been conducted examining the effectiveness of screen time interventions (including education‐focused, physical activity and restricting screen time interventions) on screen time use (Maniccia et al. 2011; Schmidt et al. 2012; Wahi et al. 2011; Wu et al. 2016; Zhang et al. 2022). However, few studies have investigated whether screen time interventions result in the reduction of obesity or adiposity. In a 16‐week‐long health education intervention targeting physical activity and screen time (Switch Off Get Active) in primary school children, Harrison et al. (2006) showed that replacement of screen time with physical activity reduced overall screentime use and reduced weight status (Harrison et al. 2006). A meta‐analysis assessing the success of screentime interventions in adolescents highlighted that education‐based approaches were less effective than those restricting screen time or those promoting physical activity at reducing screen time use and weight status (Zhang et al. 2022). However, the meta‐analyses concluded that intervention studies need a longer follow‐up time to identify if they have an impact on sleep or obesity (Zhang et al. 2022).

Late use of screen time, for example, in the hour before bed, has been associated with poorer and later pre‐sleep and increased obesity measures (Goodman et al. 2020). Blue light exposure before bed could contribute to insomnia symptoms and poorer sleep hygiene (Cabré‐Riera et al. 2019). Furthermore, adolescents have reported passively eating and drinking SSBs while using screens, contributing to increased obesity prevalence (Huo et al. 2022). Consequently, the timing of both screen time and the passive eating could be a target for interventions to improve sleep hygiene, sleep onset, bedtime routine, and reduced obesity. Rather than restricting or eliminating screen time, which could be difficult for adolescents, affecting their social and emotional wellbeing (Jones et al. 2021), allotting a specific time of day to screen time may be a more practical approach.

### 4.4 Health Determinants of Poor Sleep, Obesity and Higher Adiposity

Studies included in this systematic review that examined the health determinants of poor sleep, obesity and adiposity focused more on emotional wellbeing (depressive symptoms, anxiety, food addiction and disordered eating, psychological distress) and sleep and obesity rather than physical wellbeing. When higher‐quality studies are considered (Figure 4) emotional wellbeing has been more consistently reported to be a determinant of poor sleep and obesity than the physical wellbeing variables. Few studies met the inclusion criteria assessing physical health (Figure 4). However, of those included in this systematic review, many of the physical health associations were only significant with sleep or obesity and adiposity and thus, the findings from this review suggest that physical wellbeing should not be considered a shared determinant of poor sleep and obesity in adolescents.

#### 4.4.1 Emotional Wellbeing

All aspects of emotional wellbeing recorded (depressive symptoms, anxiety, food addiction and disordered eating, psychological distress) in studies used complex analyses, adjusted for the demographics of the participants, to determine if wellbeing was a predictor of poor sleep and obesity (Table 3). Wellbeing was identified, by the studies included in this review, as a predictor for poor pre‐sleep (insomnia symptoms and a later chronotype), poor during‐sleep (poor sleep quality) and increased adiposity and obesity.

The quality of studies investigating wellbeing as a shared determinant of sleep and obesity and adiposity was higher (84.4% of associations extracted where from high‐quality studies) (Figure 4) than those studies investigating socioeconomic and behavioural determinants of sleep and obesity (Figures 2, 3). Moreover, when considering higher‐quality studies only, 89.2% of the associations extracted from the studies for this review consistently identified wellbeing as a shared determinant of poor sleep, obesity and adiposity in adolescents (Figure 4).

#### 4.4.2 Depressive Symptoms

Depressive symptoms were the most researched health determinant of sleep, obesity and adiposity. However, many of the associations extracted for this review were from a lower‐quality study (LeMay‐Russell et al. 2021) (Figure 4). When only considering the higher‐quality studies, depressive symptoms were consistently reported as a shared health determinant of sleep, obesity and adiposity. The current findings showed that studies using complex predictor modelling found that depressive symptoms were predictors of poor sleep and obesity (Lima et al. 2020; Moore 2007) and that the relationship could even be bidirectional (Lima et al. 2020; Moore 2007). The findings of this systematic review are supported by other research investigating the longitudinal and bidirectional relationship between depressive symptoms, sleep and obesity, that suggests a bidirectional relationship between depression and insomnia symptoms (Alvaro et al. 2013), depression and poor sleep quality (Alvaro et al. 2013) and depressive symptoms and obesity (more common in girls than boys) (Mannan et al. 2016). Consequently, depressive symptoms should be considered a shared determinant of obesity and poor sleep in adolescents and, due to the bidirectional associations in previous research (Lima et al. 2020; Moore 2007) it could be suggested that poor well‐being co‐occurs with poor sleep and obesity across adolescence.

#### 4.4.3 Anxiety

All studies included in this review that assessed anxiety as a shared determinant of sleep, obesity and adiposity were of higher quality and reported that anxiety severity was a shared determinant of poorer during‐sleep and post‐sleep outcomes and higher adiposity (Lima et al. 2020; Moore 2007) (Figure 4). No studies examined the relationship between anxiety and pre‐sleep measures and adiposity or obesity; thus, research should be conducted to address this gap. However, existing research examining the relationship between anxiety and sleep and anxiety and obesity independently has indicated a bidirectional (Manzar et al. 2020) and indirect (Rakhimov et al. 2022) relationship between anxiety, sleep hygiene and bedtime routine, and between anxiety and obesity (Amiri and Behnezhad 2019). Consequently, like depressive symptoms, anxiety should be considered a determinant of sleep, obesity and adiposity measures, and further studies to assess the bidirectional nature of the relationship and whether anxiety co‐occurs with poor sleep and obesity across adolescence should be conducted.

#### 4.4.4 Psychological Stress, Suicide Ideation and Internalising Problems

Psychological stress, suicide ideation, and internalising problems were identified as shared health determinants and predictors of poor pre‐sleep (sleep hygiene), during‐sleep, and obesity in higher quality studies, including studies with more complex analyses (Lin et al. 2020; El‐Sheikh et al. 2007; El‐Sheikh et al. 2019) (Figure 4) These findings are supported by secondary data analysis from the 2019 Youth Risk Behaviour Survey (US), where the prevalence of suicide attempts was 8.90% in a healthy‐weight population compared with 15.5% in a population living with obesity (Iwatate et al. 2023). Furthermore, in a scoping review of the prevention of internalising disorders and suicide via adolescent sleep interventions, behavioural sleep interventions that improved sleep problems saw a significant improvement in depression and anxiety in adolescents (Blake and Allen 2020). Thus, mental health, sleep and obesity appear to play a role in the development of one another.

#### 4.4.5 Disordered Eating

Disordered eating was identified in systematic reviews as a shared health determinant of poorer pre‐sleep, during‐sleep and post‐sleep outcomes and increased obesity (Lin et al. 2020; Saleh‐Ghadimi et al. 2019; Parker et al. 2022; Pickett et al. 2022). The findings are supported by existing research which indicates a bidirectional relationship between binge eating, dysregulated sleep and orexin plasticity (Mehr et al. 2021). Pharmacologically targeting the orexin signalling to reduce responsiveness to food cues and increase inhibitory control could help improve sleep regulation and reduce food addiction in adolescents (Mehr et al. 2021). However, as behavioural interventions are more economically viable and acceptable than pharmacological interventions in adolescents (Zanganeh et al. 2019), a behavioural intervention targeting food consumption habits and sleep could be feasible. Nevertheless, treating disordered eating can be difficult due to those exhibiting disordered eating often having other risky behaviours (such as smoking), wellbeing concerns (such as depression and anxiety) and these individuals tend to have a higher BMI than those without disordered eating (Mehr et al. 2021; Horsager et al. 2022).

### 4.5 Interactions Between Determinants of Sleep, Obesity and Adiposity

In addition to identifying the shared determinants of poor sleep and obesity in adolescents, researchers have identified key interactions between socioenvironmental and behavioural determinants of sleep, obesity and adiposity. Gender differences have been reported in screen time (Khan et al. 2022), videogaming and social media use (Lovreković et al. 2022), “internet addiction” (Twenge and Farley 2021), diet choices (SSBs, fast food, Mediterranean diet, energy intake) (Lee and Allen 2021), physical activity (Kallio et al. 2020), depression and anxiety (Hyde and Mezulis 2020) and food addiction and disordered eating (Lin et al. 2020). Additionally, ethnicity differences have been reported in diet choices, screen time, physical activity (Delgado‐Floody et al. 2020) and depression, anxiety and stress (Daly 2022; Fox et al. 2020). Socioeconomic status has been associated with depression (Daly 2022), internalising problems (Ramos et al. 2019), SSB consumption (Männikkö et al. 2020), screen time and physical activity (Männikkö et al. 2020). Pubertal status has been associated with screen time (Hoyt et al. 2020), chronotype, depression and anxiety (Haraden 2022; Haraden et al. 2019), meaning that some of these interactions will start earlier in females than males and should be considered when designing and recruiting for interventions in this population.

Multiple interactions between behavioural and health determinants of sleep, obesity and adiposity have also been reported. Increased SSB consumption has been associated with depression (Xu et al. 2020), anxiety (stratified by adiposity) (Liu et al. 2022), food addiction (Lemeshow et al. 2018), suicide ideation (Kim et al. 2022) and chronotype (Li et al. 2018). Additionally, fast food consumption has been associated with depression (Xu et al. 2020), anxiety (Ishak et al. 2022) and suicide ideation (Jacob et al. 2020). Screen time has been associated with depression (Stiglic and Viner 2019), anxiety (Kim et al. 2020) and disordered eating (Nagata et al. 2021) and videogaming has been associated with depression (Brunborg et al. 2014) and anxiety (Brunborg et al. 2014).

Further research should be conducted to understand the complexity of interactions between determinants of poor sleep, obesity and adiposity so that they can be targeted in a multi‐component intervention.

### 4.6 Strengths and Limitations of the Systematic Review

To the authors' knowledge, this is the first review to systematically identify shared determinants of sleep, obesity and adiposity in adolescents across three categories: socioenvironmental demographics, behavioural and health. Determinants of sleep duration were not explored in this review due to this being previously reported (Hawkins and Takeuchi 2016; Hitze et al. 2009; Inhulsen et al. 2022). The findings of this review have highlighted several modifiable determinants that could be targeted for a health‐promoting intervention, and identified those non‐modifiable determinants that should be factored into the data collection and analysis of future studies assessing sleep and obesity.

Another strength of this systematic review is the age range (8–18 years) explored. By focusing on 8–18 years, this review provides a specific insight into the shared determinants of sleep, obesity and adiposity during the pubertal years. The number of studies included in this review, along with the wide breadth of study designs and samples, are additional strengths. Including studies examining both the sleep‐determinant association and the obesity‐determinant or adiposity‐determinant relationship is novel. Importantly, this enabled relative comparison of the determinants to sleep and adiposity, and obesity. More studies in this review measured obesity as an outcome rather than adiposity, highlighting the need for future research to prioritise adiposity as a key measure. Unlike BMI, which relies on height and weight, adiposity directly quantifies body fat, allowing for a more accurate exploration of the shared determinants of sleep and obesity. This approach reduces confounding and provides deeper insight into the physiological mechanisms linking sleep and metabolic health.

There are some limitations that should be acknowledged. The heterogeneity in the measurement and reporting of sleep and obesity variables, combined with the wide age range of participants spanning pre‐puberty to late adolescence, meant that conducting a meta‐analysis was not considered. These age‐related differences likely reflect developmental changes, such as the shift from parental control in younger children to greater independence in older adolescents, which may influence modifiable factors. The variation in confounders considered in statistical analyses across studies meant that the comparability of studies was limited. Studies examining modifiable behavioural determinants of sleep, obesity and adiposity have lacked consistency in adjusting for socioenvironmental determinants. The most common socioenvironmental determinant adjusted for in the statistical analysis of behavioural determinants was gender, followed by age, then ethnicity, SES and caregivers' education. Future research should consider confounders such as pubertal status and other caregiver demographics (such as BMI) when measuring associations of sleep, obesity and adiposity. Moreover, research to further understand the shared behavioural and health determinants of sleep, obesity and adiposity (those indicated in this review, and additional novel determinants) is warranted. Additionally, longitudinal studies and interventions should be a focus for future research. Finally, we acknowledge that this systematic review examined the shared determinants of poor sleep and obesity using a data‐driven, iterative approach based on the determinants measured in the included studies. Applying a theoretical framework could further enhance the conceptual depth of our analysis.

## 5 Conclusion

A range of shared socioenvironmental determinants (gender, ethnicity, pubertal status, academic attainment), behavioural determinants (timing of MVPA, unhealthy diet choices and timing of consumption, and screen time and videogaming quantity and timing) and health determinants (wellbeing) of sleep and obesity or adiposity in adolescents aged 8–18 years have been identified. Future research should include cross‐sectional and longitudinal studies of timing and regularity of screen time, unhealthy dietary choices, MVPA and the associations with sleep, obesity and adiposity. Due to the clustering of determinants, combined interventions targeting sleep hygiene and modifiable behavioural determinants (including regulation and timing of healthy diet choices, MVPA, screen time and videogaming), monitoring other shared health determinants (including wellbeing) and adjusting for shared socioenvironmental determinants in the statistical analysis should be conducted.

## Author Contributions

**Emma Louise Gale:** conceptualization, methodology, project administration, writing – review and editing, writing – original draft, investigation, formal analysis. **Joanne Elizabeth Cecil:** conceptualization, methodology, writing – review and editing, supervision. **Andrew James Williams:** methodology, conceptualization, supervision, writing – review and editing.

## Conflicts of Interest

The authors declare no conflicts of interest.

## Data Availability

Data sharing not applicable to this article as no datasets were generated or analysed during the current study.
